# Evaluating the effectiveness of open-weight LLMs for multi-domain Bengali text classification with zero-shot and few-shot prompting

**DOI:** 10.1371/journal.pone.0361368

**Published:** 2026-11-06

**Authors:** Md. Hamid Hosen, Sadia Nawar, Rituparna Chowdhury, Altaf Uddin, Riadul Islam Rabbi, Khair Razlan Othman, Nurul Karim Symon

**Affiliations:** 1 Department of Computer Science and Engineering, East Delta University, Chattogram, Bangladesh; 2 Faculty of Engineering and Technology, Multimedia University, Melaka, Malaysia; 3 Centre for Advanced Analytics, COE for Artificial Intelligence, Faculty of Engineering and Technology (FET), Multimedia University, Melaka, Malaysia; 4 Department of Computer Science and Engineering, International Islamic University Chittagong, Chattogram, Bangladesh; VIT Bhopal University, INDIA

## Abstract

Large Language Models (LLMs) have advanced multilingual NLP significantly, yet their effectiveness for Bengali, a globally important but low-resource language, remains understudied due to limited linguistic resources and the absence of a standardized evaluation benchmark. This is due to the unavailability of large linguistic resources and the lack of a standard benchmarking framework, which has yet to be established. This study aims to address the underrepresentation of Bengali text classification by leading a systematic evaluation. It involves assessing multiple open-weight LLMs on Bengali text classification tasks and observing their limitations and regular error patterns. The procedure consists of six open-weight LLMs, which include LLaMA-3.2-3B, Mistral-V3-7B, DeepSeek-R1-8B, Phi-4-14B, Gemma-2-27B, and Qwen-2.5-72B, and are evaluated using four critical text classification tasks in Bengali, namely sentiment analysis, emotion recognition, hate-speech detection, and fake-news classification. Furthermore, two prompting methods are compared in the context of zero-shot and few-shot prompting using both the vLLM inference engine and Hugging Face Transformers pipelines to investigate the adaptability of the models in resource-limited settings. Across all four tasks, the two largest models (Qwen-2.5-72B and Gemma-2-27B) obtained the highest scores, although the ranking was not strictly monotonic in parameter count and, because each scale is represented by a single model family, the design cannot attribute these differences to model size alone. When training in zero-shot, Qwen-2.5-72B showed the best performance at 79.90% accuracy and 79.88% Macro-F1 score, showing amazing accuracy on hate-speech detection. In the case of fake news, Gemma-2-27B performed best at 84.45% accuracy and 84.33% Macro-F1 score. With a few-shot set-up, Gemma-2-27B and Qwen-2.5-72B demonstrated a high level of performance in terms of Macro-F1 score, reaching 84.17% for fake-news detection and 66.80% for emotion recognition, respectively. Paired multi-model tests (Cochran’s Q with Holm-corrected McNemar post-hoc comparisons) confirmed significant differences among the six models across all tasks and prompting settings, whereas the effect of five-shot prompting, obtained with a single fixed demonstration set, was significant for only 4 of 24 model–task combinations after correction and never for the three largest models. The findings characterise benchmark classification performance under the evaluated settings rather than real-world effectiveness, and indicate that model scale, multilingual pre-training, and in-context demonstrations are jointly associated with, but cannot be causally isolated as drivers of, performance on Bengali text classification.

## Introduction

Large Language Models (LLMs) have revolutionized natural language processing. Large Language Models (LLMs) facilitate enhanced efficiency on different types of tasks, without large-scale training related to the task. The models utilize context-based learning to comprehend the pattern from the given prompts, which in turn decreases the requirement for a large labelled dataset. Nevertheless, it is notable that most achievements of LLMs are observed in high-resource languages as English, while the low-resource languages like Bengali do not receive enough attention. Bengali is a rich language with immense diversity, even though it is considered to be the 7th most spoken language in the world, and there are over 230 million Bengali speakers; this widely spoken language is still underrepresented [1]. In [2,3], authors mention that proprietary models like GPT-4 and Claude-3.5 present moderate capabilities in processing the Bengali language. On the other hand, open-weight models like Gemma-2 and LLaMA-3.1 show disappointing outcomes in the same task.

The main drawbacks are data quality and the substandard performance of well-known robust LLMs. The majority of the Bengali language processing conducted previously depends heavily on the machine-generated translated datasets like Bangla-Alpaca or Bangla-Orca translated version of the original Stanford Alpaca and OpenOrca dataset [4], hence it causes a rise in various errors and poor performance from the models [5]. Moreover, an extensive review shows that well-known robust LLMs encounter difficulties just to reach satisfactory outcomes in Bengali tasks. BenLLM-Eval finds that from time to time, GPT-3.5 and Claude models’ performances match the performance of fine-tuned Bengali models on a few tasks [6]. Furthermore, in the case of zero-shot, the performance of the majority of LLMs is substantially inadequate, and open-weight models (like LLaMA-2-13B) perform worse.

In case of accuracy, sizeable differences are observed between English and Bengali text-related tasks, which is more significant for smaller model series (e.g., Mistral) [7]. This difference in accuracy can be overcome via in-context learning. [8] shares that by presenting a few suitable samples, the LLMs showcase significantly enhanced understanding of the low-resource languages. Nonetheless, the research comprising Bengali language-related LLMs is still scarce.

Recently, researchers have taken the initiative to resolve the accuracy difference that LLMs face by building exclusive Bengali language resources. [9] presented the first substantial pretrained Bengali LLMs named TituLLMs. The authors also introduced five new Bengali baseline datasets and showed that the performance of Bengali-specific models is far better than that of other language models for native tasks. TigerLLM introduced in the study [2], trained on a meticulously refined Bengali dataset, showcases a new benchmark for Bengali language processing, surpassing the previous open-weight models. GPT-3.5 Turbo achieved more than 97% accuracy in Bengali hate speech detection by utilizing few-shot prompting, which outperforms fine-tuned encoder-based transformer models [10]. Regardless of the mentioned accomplishments, there is still a limited number of extensive multi-domain baseline and systematic prompt assessments. The majority of previous research focuses on individual tasks or utilizes translated English baselines, which led to a rise in noise [5,7]. Confronting these differences in outcomes is vital for creating a multilingual natural language processing model. The main objective of our research is to provide a systematic evaluation of open-weight LLMs based on four Bengali text classification tasks under zero-shot and few-shot prompting conditions.

According to a few recent studies, model dimension and the scope of the training dataset have an immense influence on the downgrade of performance [11]. In a study [12], bigger models show amazing performance in zero-shot tasks for high-resource languages like English; however, the performance plummets for low-resource languages. And many LLMs can learn and solve problems with the help of few-shot prompting [13]. [8] reveals that introducing a few samples via few-shot prompting helps enhance the model performance across 25 undervalued languages. However, the Bengali language is not specifically included in this list of undervalued languages. Because of the lack of large-scale pre-trained LLMs focused on the Bengali natural language processing domain, most advancements rely heavily on smaller fine-tuned encoder models like BanglaBERT [14]. During the research, [7] found that current multilingual datasets consist of around 30 billion Bengali text tokens; in contrast, the English language consists of a trillion tokens, which results in poor model quality. In [6], build BenLLM-Eval, a baseline model capable of summarization, classification, and QA tasks in the Bengali language. Additionally, they notice that even though GPT-3.5 sometimes outperforms the fine-tuned models, its performance is poor for a zero-shot system. Likewise, [9] introduced TituLLMs and native baselines, showcasing enhanced performances across multilingual models. Regardless of these initiatives, significant research gaps are still present. Bengali language classification has not been analyzed appropriately via zero-shot and few-shot settings. Latest open-weight models, namely Qwen, Mistral, or Phi-4, have yet to be evaluated on Bengali text classification tasks, and overall error assessment of the models is lacking. To tackle these research gaps, our study aims to provide a systematic evaluation of six open-weight LLMs across four Bengali text classification tasks with further insight into their limitations and error patterns.

To analyze the performance of open-weight LLMs for Bengali text classification tasks, this study is guided by the following research questions:

- **RQ1:** What is the performance of open-weight LLMs on Bengali text classification over several different domains, consisting of sentiment analysis, emotion recognition, hate speech detection, and fake news detection?
- **RQ2:** How are differences in model scale and multilingual pre-training characteristics associated with Bengali text classification performance across the evaluated models?
- **RQ3:** How does five-shot prompting affect accuracy, recall, and Macro-F1 compared with zero-shot prompting across the evaluated Bengali text classification tasks?
- **RQ4:** What sort of incorrect classification, limitations, or systemic errors are usually found during LLMs performance for Bengali text tasks, and what type of limitations are stipulated by these error patterns?
- **RQ5:** How uniform are various open-weight LLMs in classifying diverse Bengali natural language processing tasks, when assessed using a unified, prompt-based pipeline?

This research presents several noteworthy contributions to the rising field of the Bengali natural language processing domain:

1. **Comprehensive Evaluation:** We perform a comprehensive evaluation of six of the latest open-weight LLMs: LLaMA-3.2-3B, Mistral-V3-7B, DeepSeek-R1-8B, Phi-4-14B, Gemma-2-27B, and Qwen-2.5-72B, on four types of Bengali text classification tasks: sentiment analysis, emotion recognition, hate speech detection, and fake news classification.
2. **Unified Prompting Framework:** We apply a systematic evaluation pipeline utilizing both the vLLM inference engine and Hugging Face Transformers pipelines, while using zero-shot and few-shot prompting to maintain consistency and reproducibility over the models’ experiments.
3. **Performance Insights:** The two largest models (Qwen-2.5-72B and Gemma-2-27B) generally achieve the strongest performance across the evaluated tasks. Because model scale, pre-training data, tokenizer, and instruction tuning vary jointly across the evaluated model families, these results are reported as associations rather than as causal effects of model size.
4. **In-Context Learning Effects:** Five-shot prompting with a single fixed demonstration set produces task- and model-dependent effects, including improvements and occasional degradation among smaller models, while showing no statistically significant change for the three largest models. We therefore describe these effects as changes in classification performance under the evaluated Bengali task settings rather than as improvements in Bengali language understanding.
5. **Detailed Error and Class-Wise Analysis:** We utilized comprehensive evaluation metrics (Accuracy, Precision, Recall, Macro-F1) and confusion matrices, complemented by bootstrap confidence intervals for all models and a quantitative error-category analysis (confusion-pair shares, false-negative/false-positive asymmetry, and negation-marker and surface-cue statistics). This enables recognizing common model errors and recommending an approach for future improvements.

The paper is organized into the following sections: First, the Related Work section presents a literature review of prior research on Large Language Models (LLMs), multilingual natural language processing, and Bengali text classification. The Methodology section provides a detailed explanation of the chosen models, dataset preparation, experiment structure, and application of zero-shot and few-shot prompting. The Results section reports the overall performances of the models using evaluation metrics for four types of Bengali text classification tasks, and also presents a comparison among the chosen models. The Discussion section provides detailed insights into the performance patterns, overall findings, common mistakes, and errors related to misclassification. Lastly, the Conclusion section summarizes the contribution of the research, notes the limitations, and recommends future direction of work.

## Related work

Large Language Models (LLMs) for the Bengali language are currently gaining growth, similar to previous advances in Natural Language Processing. Though Bangla is one of the most widely spoken languages in the world, it still lags behind in the field of computational linguistics due to limited annotated corpora, ineffective tokenization, and inadequate domain coverage. In recent times, the focus has been on model adaptation, resource generation, and zero- or few-shot prompting strategies to improve Bengali text understanding and classification across multiple domains.

### Progress in Bengali LLMs and benchmark development

Recent years have witnessed substantial progress in Bengali natural language processing (NLP), driven by the development of Bengali-specific pre-trained language models, multilingual large language models (LLMs), benchmark datasets, and evaluation frameworks. Table 1 summarizes representative studies and their key contributions to Bengali NLP research.

**Table 1:** Summary of recent studies on Bengali LLMs, benchmarks, and text classification.

Reference	Model	Task / Domain	Key Findings
Dehan et al. [15]	Gemma-2B, TinyLLaMA, Falcon, BanglaBERT	Bengali text classification	TinyLLaMA achieved the best performance for fake news detection, while BanglaBERT excelled in emotion recognition.
Rahman et al. [16]	BERT, RoBERTa	Sentiment analysis	Achieved 88% accuracy on Bengali movie reviews, demonstrating the superiority of transformer-based models over traditional machine learning approaches.
Kabir et al. [6]	BenLLM-Eval	Bengali LLM evaluation benchmark	Introduced a benchmark framework for evaluating GPT-3.5, Claude-2, and LLaMA-2 across diverse Bengali NLP tasks.
Bhattacharjee et al. [14]	BanglaBERT	Bengali language modeling	Developed a Bengali-specific language model trained on 27 GB of text and demonstrated strong performance in sentiment analysis and NER.
Mahmud et al. [17]	Hybrid BanglaBERT framework	Sentiment analysis	Combined lexicon-based scoring with BanglaBERT embeddings and achieved an F1-score of 93%.
Bhowmik et al. [7]	Qwen, Mistral, DeepSeek	Bengali benchmark evaluation	Reported that tokenization issues and translation noise remain major obstacles for multilingual LLMs.
Al Nazi et al. [18]	GPT-4o, Gemini, BLOOM	Zero-shot and few-shot evaluation	Compared open- and closed-source models and highlighted the rapid progress of prompting-based approaches.
Prome et al. [19]	Metaphor-based prompting	Hate speech detection	Improved interpretability and fairness in Bengali hate speech classification through metaphor-guided prompting.
Banda et al. [20]	Few-shot multilingual LLMs	Agricultural question answering	Demonstrated strong generalization capabilities under limited supervision.
Raihan and Zampieri et al. [2]	TigerLLM	Bengali instruction-tuned LLM	Released an open-source Bengali LLM family with performance comparable to GPT-3.5 on several tasks.
Mahfuz et al. [21]	Bengali-specific LLM analysis	Bengali NLP	Highlighted the importance of language-specific pre-training for capturing Bengali linguistic and cultural nuances.

As summarized in Table 1, prior studies have explored Bengali-specific pre-trained language models, multilingual LLM evaluation, benchmark construction, prompting strategies, and task-specific applications such as sentiment analysis, hate speech detection, and question answering. These efforts collectively demonstrate the growing maturity of Bengali NLP research while also revealing persistent challenges related to tokenization, contextual understanding, and culturally grounded language phenomena. Motivated by these developments, the present study provides a comprehensive evaluation of six contemporary open-weight LLMs across four Bengali text classification tasks under both zero-shot and few-shot prompting settings.

### Resource creation and cross-domain studies

Mahtab *et al.* [22] developed BanNERD, a comprehensive 29-domain Bengali NER dataset intended to improve contextual generalization in named-entity recognition algorithms. In a similar vein, Shafayat *et al.* [23] published BEnQA, a bilingual Bengali-English question-answering dataset that demonstrated considerable thinking differences between the two languages. Singh *et al.* [24] further multilingual research by developing IndicGenBench, a generative benchmark encompassing 11 Indic languages, indicating that contemporary multilingual systems still face significant hurdles in addressing morphological and syntactic variances. Shukla *et al.* [25] introduced IndicMMLU-Pro, a multilingual reasoning benchmark, and found that Indic LLMs continue to lag English-centric models in comprehension tests. Expanding resources for Bengali NLP. Fahim *et al.* [26]presented BanglaTLit, a transliteration dataset with 42.7K Romanized Bengali items, in which fine-tuned encoder models reached cutting-edge performance. Haider *et al.* [27] introduced BANTH, a multi-label hate-speech dataset for transliterated Bengali that significantly increased zero-shot classification accuracy. Romim *et al.* [28] assembled BD-SHS, a dataset of 50,000 online hate-speech samples, and achieved a 91% F1-score using contextual learning approaches. Nayeem *et al.* [29] proposed MultiBanFakeDetect, a multimodal approach for detecting false news that combines textual and visual data to improve cross-domain robustness. Furthermore, Hasan *et al.* [30] developed Bangla_ABSA, the first aspect-based sentiment analysis dataset for Bengali, which covers domains such as restaurants, mobile devices, and autos, allowing for fine-grained polarity recognition.

Nahin *et al.* [9] continued the advancement in large-scale Bengali modeling by introducing TituLLMs, a family of Bengali large language models trained on 37 billion tokens that outperformed multilingual models such as LLaMA and Zephyr in reasoning and comprehension tasks. Faria *et al.* [10] compared GPT-3.5 Turbo and Gemini 1.5 Pro to transformer-based baselines for hate-speech recognition and achieved 98.5% accuracy in a few shots. Hasan *et al.* [31] explored zero-shot and few-shot prompting for sentiment analysis and discovered that in-context examples greatly improve LLMs’ generative capacities. Rafid *et al.* [32] compared decoder-only and encoder-based models for multi-label document categorization and concluded that decoder architectures respond better to unstructured textual input. Likewise, Saha *et al.* [33] established a hybrid knowledge-driven model that identifies altered Bengali news using language signals and semantic embeddings, with an accuracy of more than 90%.

### Domain-specific LLM adaptations and applications

Hossain *et al.* [34] proposed BanglaNewsClassifier, a hybrid stacking ensemble that uses SVM, Random Forest, and XGBoost models to classify 118K Bengali news articles with 94% accuracy. Chowdhury *et al.* [35] demonstrated DepGPT, a fine-tuned large language model designed to detect depressive language in social media content, with an accuracy of 97.9%, demonstrating how LLMs may efficiently capture emotional and psychological patterns in text. Kamruzzaman *et al.* [5] generated BanMANI, an annotated dataset for detecting altered Bengali social media news, underlining the significance of domain-specific data resources for misinformation identification. Faria *et al.* [3] evaluated the performance of large language models (GPT-3.5, Gemini 1.5 Pro) and transformer-based architectures (BanglaBERT, DistilBERT) on Bengali natural language inference, revealing significant variations in reasoning capacities. Their findings showed that few-shot prompting considerably improved reasoning abilities over standard fine-tuning methods. Maity and Deroy *et al.* [36] investigated prompt-tuning strategies for Bengali grammatical error explanation using GPT-4, GPT-3.5, and LLaMA-2 and found significant increases in both accuracy and interpretability, indicating a potential direction for educational NLP applications in Bengali.

Overall, this research show Bengali NLP’s rapid progress in model construction, dataset development, and cross-domain evaluation. Earlier works were primarily concerned with resource construction and baseline transformer models; however more current research has switched to prompt engineering, multilingual adaptability, and task-specific implementations. Despite these developments, Bengali NLP continues to confront issues like as data asymmetry, inadequate tokenization, and a lack of fine-tuning resources. Building on these advances, the current study conducts a thorough evaluation of open-weight LLMs for multi-domain Bengali text categorization under zero-shot and few-shot prompting scenarios, revealing novel insights into scalable and resource-efficient modelling for low-resource languages.

## Methodology

This research performs four types of Bengali text classification utilizing six open-weight Large Language Models (LLMs). The six LLMs include LLaMA-3.2-3B (Meta AI), Mistral-V3-7B (Mistral AI), DeepSeek-R1-8B (DeepSeek), Phi-4-14B (Microsoft), Gemma-2-27B (Google DeepMind), and Qwen-2.5-72B (Alibaba Cloud). The four Bengali text classification tasks were sentiment analysis, emotion recognition, hate speech detection, and fake news detection.

The selected LLMs represent a diverse range of model sizes, pre-training corpora, and post-training optimization strategies. Although all evaluated models are based on Transformer decoder architectures, they differ in parameter scale, multilingual capabilities, instruction-tuning procedures, and alignment techniques. Some models additionally employ reinforcement learning-based post-training to enhance reasoning and instruction-following performance. This diversity enables a comprehensive comparison of open-weight LLMs for Bengali text classification.

In the rest of the paper we use the short model names that appear in the tables and figures: LLaMA-3.2-3B, Mistral-V3-7B, DeepSeek-R1-8B, Phi-4-14B, Gemma-2-27B and Qwen-2.5-72B. Each short name refers to exactly one Hugging Face checkpoint, and the full repository identifiers are given in the Models subsection. We also prefer the term *open-weight* to *open-source*. The weights of all six models can be downloaded freely, but some of them, such as the Llama and Gemma families, come with licences that do not meet the Open Source Definition.

The methodology of this research is presented in the diagram, Fig 1. Initially, the Bengali text classification datasets were prepared from multiple domains, which include sentiment analysis, emotion recognition, hate speech detection, and fake news detection. The models separately undergo a zero-shot and a few-shot experimental setup. It mainly helps understand the models’ in-context learning ability. The models were provided with the input sample and task instructions in case of the *zero-shot* setup. And for the *few-shot* setup, before the test input, five labeled Bengali text samples were added to the prompt, enabling the model to determine contextual relationships. The purpose of this setup is to assess whether the sample-based context enhances the performance for low-resource languages.

**Fig 1:**
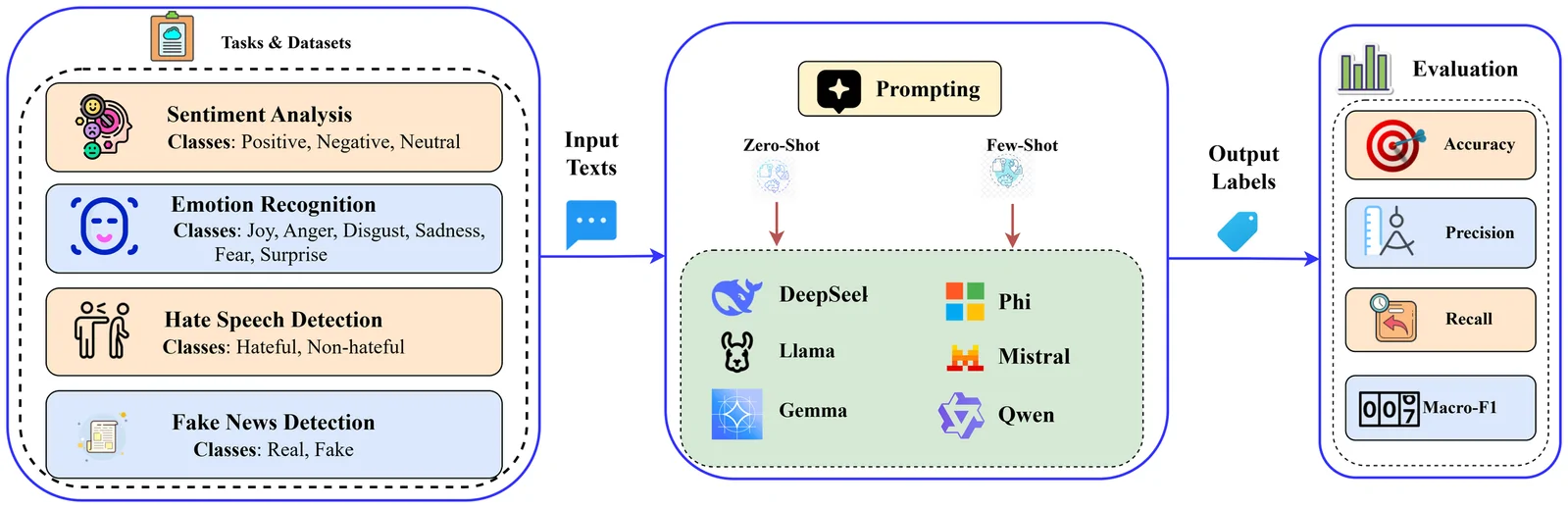
Overall methodology illustrating the evaluation framework for six open-weight LLMs across four Bengali text classification tasks under zero-shot and few-shot prompting.

### Datasets and tasks

This research aims to evaluate Large Language Models based on their performance in Bengali text classification. To properly assess the models, four types of text classification were divided into two categories: opinion-based judgment (sentiment analysis and emotion recognition) and fact-based verification judgment (hate speech detection and fake news detection). The detailed information on the datasets is displayed in Table 2.

**Table 2:** Summary of datasets used in this study.

Task	Dataset	Classes	Test Size (Samples)
Sentiment Analysis	SentiNoB [37]	3	1,586
Emotion Recognition	BEmoC [38]	6	625
Hate Speech Detection	Bengali HS [39]	2	1,000
Fake News Detection	BanFakeNews [40]	2	508

#### Sentiment analysis.

The SentiNoB [37] dataset was used for sentiment analysis. This dataset consists of three categories collected from social media and annotated into positive, negative, and neutral. The categories contain 1586 samples, where positive was 654, negative was 571, and neutral was 361.

#### Emotion recognition.

The Bangla Emotion dataset [38] introduced by Das *et al.* was used for emotion recognition analysis. The dataset was annotated into six categories: joy, anger, disgust, sadness, surprise, and fear. The dataset was gathered from social networking sites and annotated via a crowd-sourcing platform. The test set comprises 625 samples and is highly imbalanced across the classes (e.g., 165 samples for disgust versus only 71 for anger), reflecting the natural distribution of emotional text on social media.

#### Hate speech detection.

The Bengali Hate Speech Dataset [39] was used to evaluate the hate speech detection capability of models. The binary classification framework was implemented for uniformity. A balanced dataset proportion can be viewed in the test set, which consists of 500 samples of hateful and 500 samples of non-hateful samples.

#### Fake news detection.

Fake news detection was assessed using the BanFakeNews dataset [40]. The dataset consists of approximately 20,000 real news articles and 254 fake news articles. Since the dataset is highly imbalanced, the majority class (real news) was randomly downsampled using a fixed random seed of 42 to ensure reproducibility. Specifically, all 254 fake news samples were retained, and 254 real news samples were randomly selected from the majority class, resulting in a balanced evaluation set of 508 samples with a 1:1 class ratio. This balancing changes the evaluation distribution: fabricated articles make up only about 1.3% of the source corpus (254 of roughly 20,254 articles) but 50% of our evaluation set. The scores for this task therefore describe performance under a balanced class prior, and the precision of the fake class in particular would be much lower at the natural class distribution. We avoided oversampling methods such as text duplication or synthetic data generation to prevent introducing artificial patterns into the evaluation. Since the evaluation set was constructed solely from existing samples, no textual content was modified during the data preparation process. However, we acknowledge that random downsampling may not fully preserve the semantic distribution of the original dataset, which represents a potential limitation of this evaluation setting. Despite this limitation, the balanced test set provided a practical and consistent setting for comparing the performance of different LLMs under identical evaluation conditions.

### Models

In this research, experiments were conducted on six Large Language Models (LLMs). The models were selected based on variations in model size, training data, and optimization strategies. The evaluated models range from smaller 3B-parameter systems to larger multilingual 72B-parameter systems. Although all evaluated models are based on Transformer decoder architectures, they differ in their pre-training corpora, parameter scale, and post-training alignment methods. In particular, some models employ additional optimization techniques, such as reinforcement learning-based techniques, to improve reasoning and instruction-following capabilities. These differences provide a diverse basis for evaluating Bengali text classification performance.

To ensure full transparency and reproducibility of our experiments, we utilized the official model weights hosted on the Hugging Face Hub. Specifically, the evaluated models correspond to the following repository identifiers: meta-llama/Llama-3.2-3B-Instruct, mistralai/Mistral-7B-Instruct-v0.3, deepseek-ai/DeepSeek-R1-Distill-Llama-8B, microsoft/phi-4, google/gemma-2-27b-it, and Qwen/Qwen2.5-72B-Instruct-AWQ (all instruction-tuned versions; models and tokenizers were loaded from the default revision of each repository, and each model’s own chat template was used without modification). Notably, for the Qwen-2.5-72B model, we employed the AWQ (Activation-aware Weight Quantization) version to fit the model within the available VRAM constraints while maintaining robust inference performance.

The overall specifications of the evaluated models are summarized in Table 3.

**Table 3:** Overview of evaluated large language models.

Model	Parameters	Architecture	Developer
LLaMA-3.2-3B	3B	Transformer	Meta AI
Mistral-V3-7B	7B	Transformer	Mistral AI
DeepSeek-R1-8B	8B	Transformer	DeepSeek
Phi-4-14B	14B	Transformer	Microsoft
Gemma-2-27B	27B	Transformer	Google DeepMind
Qwen-2.5-72B	72B	Transformer	Alibaba Cloud

The six selected models for assessment follow the decoder-only Transformer paradigm introduced by Vaswani et al. [41] and later scaled successfully in GPT-style language models such as GPT-3 [42], where the self-attention mechanism is the central component.

The computation of the self-attention mechanism for the input sequence is as follows:


$$\begin{array}{c} \text{Attention}(Q,K,V)=\text{softmax}\left(\frac{QK^{T}}{\sqrt{d_{k}}}\right)V \end{array}$$ (1)

Here, *Q* denotes query, *K* denotes key, and *V* denotes value metrics, and the key-vector dimension is $d_{k}$. This is improved by a multi-head attention mechanism, through the computation of attention in diverse representational layers:


$$\begin{array}{c} \text{MultiHead}(Q,K,V)=\text{Concat}({\text{head}}_{1},...,{\text{head}}_{h})W^{O} \end{array}$$ (2)

Here, the single head computes as:


$$\begin{array}{c} {\text{head}}_{i}=\text{Attention}(QW_{i}^{Q},KW_{i}^{K},VW_{i}^{V}). \end{array}$$ (3)

Table 4 outlines the characteristics of six LLMs, including the architectural specification, the depth of layers, training parameters, and hidden dimensions.

**Table 4:** Detailed architectural specifications of evaluated large language models.

Model	Layers (L)	Attention Heads (h)	Hidden Dim ($d_{h}$)	Context Length	Vocab Size	Training Tokens
LLaMA-3.2-3B	28	28	3,072	8,192	128,256	15T
Mistral-V3-7B	32	32	4,096	32,768	32,000	7T
DeepSeek-R1-8B	32	32	4,096	4,096	128,256	2T
Phi-4-14B	40	32	5,120	16,384	32,064	1.5T
Gemma-2-27B	46	32	4,608	8,192	256,128	13T
Qwen-2.5-72B	80	64	8,192	131,072	151,936	18T

#### LLaMA-3.2-3B.

The LLaMA(Large Language Model Meta AI) series was developed by Meta [43]. It provides open and effective models for the use of multiple languages. In this series, the smallest version is the 3.2B version, and it is the most compact model with competence suitable for multiple languages, and for environments with limited resources. LLaMA-3.2-3B consists of a conventional transformer decoder structure with 28 layers. These individual layers include multi-head self-attention, which is accompanied by a feed-forward network.

A variation of multi-head attention named Grouped-Query Attention (GQA) [44] is implemented in this model. Grouped-Query Attention (GQA) preserves the multiple query heads, while reducing the number of key–value heads. The mathematical expression is as follows:


$$\begin{array}{c} \text{GQA}(Q,K,V)=\text{Concat}({\text{head}}_{1},...,{\text{head}}_{h})W^{O} \end{array}$$ (4)

While the concatenation resembles standard multi-head attention, GQA organizes the *h* query heads into *g* groups (where *g* < *h*). Each group shares a single key and value head. For LLaMA-3.2-3B, these queries consist of 28 query heads and 4 key-value bundles. This design decreases the memory requirements during the predictions, while ensuring the accuracy of the model, just like a full multi-head attention.

The SwiGLU activation function used in each layer of the feed-forward network is as follows:


$$\begin{array}{c} \text{FFN}(x)=(\sigma (xW_{1})⊙xW_{2})W_{3} \end{array}$$ (5)

Here, σ(x)=x·sigmoid(x), where σ refers to the SiLU (also known as Swish) activation function, and ⊙ refers to the element-wise multiplication. The dimension of intermediate feed-forward is $d_{ff}=8192$, which is almost $2.67\times d_{h}$.

LLaMA-3.2-3B was pre-trained on approximately 15 trillion text units from a huge multilingual text compilation, which covers more than 100 languages. The model utilizes the Byte-Pair-Encoding (BPE) tokenizer, which consists of 128,256 tokens maintaining the proper handling of complex scripts like Bengali. For positional encoding, the model uses Rotary Position Embeddings (RoPE), as follows:


$$\begin{array}{c} \text{RoPE}(x_{m},m)=[\begin{array}{cc} \cos(m\theta ) & -\sin(m\theta )\\ \sin(m\theta ) & \cos(m\theta ) \end{array}][\begin{array}{c} x_{m}^{\left(1\right)}\\ x_{m}^{\left(2\right)} \end{array}] \end{array}$$ (6)

Here, *m* denotes the position index and θ denotes the frequency parameter. This encoding improves the model’s capability to scale to longer sequences.

The overall parameter count is approximately as follows:


$$\begin{array}{c} N_{\text{params}}=L(4d_{h}^{2}+2d_{h}d_{ff})+Vd_{h}\approx 3.2\times 10^{9} \end{array}$$ (7)

Here, *L* refers to 28 layers, $d_{h}=3072$ refers to 3072 hidden dimensions, $d_{ff}=8192$ refers to 8192 feed-forward dimensions, and *V* refers to 128,256 vocabulary size. Even though LLaMA-3.2-3B is on the smaller side, it still displays remarkable performance, making it ideal for zero-shot and few-shot multilingual evaluation.

#### Mistral-V3-7B.

Mistral is a lightweight and effective large language model created by MistralAI; it contains 7 billion parameters [45]. The architecture is designed in such a way that it balances the prediction speed, reasoning capability, and computational efficiency. The original Mistral structure was improved with multiple enhancements to generate the third-generation model, Mistral-V3-7B. The most notable improvement was the Sliding Window Attention (SWA) mechanism, which helps with the effective processing of lengthy sequences.

The Sliding Window Attention (SWA) mechanism restricts each token’s attention computation to a specific window of size *W*:


$$\begin{array}{c} \text{SWA}(Q,K,V{)}_{i}=\text{softmax}\;\left(\frac{Q_{i}K_{\left[i-W:i+W\right]}^{T}}{\sqrt{d_{k}}}\right)V_{\left[i-W:i+W\right]} \end{array}$$ (8)

Here, the position index is *i*, and [i−W:i+W] refers to the adjacent tokens that are near *W*. Mistral utilized *W* (4096), maintains local coherence while enabling effective long-context modeling. This helps decrease the processing complexity from *O*(*n*^2^) to O(n·W), the *n* refers to the length of the sequence.

The structure is made up of 32 Transformer layers, a single layer contains 32 attention heads and 4096 hidden dimensions. The SwiGLU activation is implemented in the feed-forward network, where the intermediate dimension is as follows:


$$\begin{array}{c} d_{ff}=14,336\approx 3.5\times d_{h} \end{array}$$ (9)

Rotary Positional Embeddings (RoPE) are configured for the coherence with the sliding window pattern. The tokenizer consists of 32,000 tokens, which are built on parts of sentences. Initially augmented for the European languages, with slight inclusion of South Asian languages.

Around 7 trillion tokens were used to pre-train the base model; these tokens were gathered from various sources such as academic texts, code, and web documents. The model evaluated in this study corresponds to the instruction-tuned variant, Mistral-7B-Instruct-v0.3. Following the initial pre-training stage, this variant underwent additional instruction tuning and alignment procedures to improve instruction-following and response quality [45]. These post-training stages enhance the model’s ability to generate more accurate and contextually appropriate responses.

#### DeepSeek-R1-8B.

DeepSeek AI introduced the DeepSeek-R1 family to significantly advance reasoning capabilities, particularly for complex tasks involving mathematics, coding, and STEM problem-solving [46]. The specific model evaluated in this study is officially named DeepSeek-R1-Distill-Llama-8B, an 8-billion-parameter distilled model built upon the Llama-3.1-8B architecture. This variant inherits advanced reasoning capabilities distilled from the larger DeepSeek-R1 model while maintaining a significantly smaller computational footprint.

The architecture of DeepSeek-R1-Distill-Llama-8B follows a standard dense Transformer decoder design consisting of 32 attention heads, 32 hidden layers, and a hidden dimension of 4096. Therefore, reinforcement learning is not considered an architectural component, but rather a post-training optimization and alignment technique used to improve reasoning performance. The model employs self-attention mechanisms, feed-forward networks, Rotary Positional Embeddings (RoPE), and RMSNorm for efficient language modeling.

The model is initially trained using causal language modeling through next-token prediction:


$$\begin{array}{c} {ℒ}_{\text{LM}}=-\sum_{t=1}^{T}\log P(x_{t}|x_{<t};\theta ) \end{array}$$ (10)

where $x_{t}$ denotes the token at step *t*, *x*_< *t*_ represents the preceding *t*okens, and θ denotes the model parameters.

The full DeepSeek-R1 training pipeline incorporates reinforcement learning, specifically Group Relative Policy Optimization (GRPO), to enhance reasoning abilities. The distilled model evaluated in this study inherits reasoning patterns learned through this process. The optimization objective is expressed as:


$$\begin{array}{c} {ℒ}_{\text{RL}}=-{𝔼}_{y\sim \pi_{\theta }(y|x)}\left[r(x,y)-\beta \;KL\left(\pi_{\theta }(y|x)\|\pi_{ref}(y|x)\right)\right] \end{array}$$ (11)

where *r*(*x*,*y*) denotes the reward assigned to output *y* for input *x*, $\pi_{\theta }$ is the policy being optimized, $\pi_{ref}$ is the reference policy, and β controls the divergence penalty. The reward mechanism consists of accuracy rewards and format rewards, encouraging both correct answers and structured reasoning outputs.

The model employs Rotary Positional Embeddings (RoPE) for positional encoding and RMSNorm for normalization:


$$\begin{array}{c} \text{RMSNorm}(x)=\frac{x}{\text{RMS}(x)}\cdot \gamma ,\;\text{RMS}(x)=\sqrt{\frac{1}{d}\sum_{i=1}^{d}x_{i}^{2}} \end{array}$$ (12)

where γ is a trainable scaling parameter and *d* denotes the dimensionality of the hidden representation.

#### Phi-4-14B.

The Phi models are generated by Microsoft to focus on the quality of data and compilation, along with scope of data [47]. The Phi-4-14B models are a combination of size and computational efficacy. Additionally, this model presents noteworthy outcomes in structured identification and reasoning problems. The structure of Phi-4-14B consists of a conventional Transformer design, which includes 40 layers, 32 attention heads and 5120 hidden dimensions.

The principal alteration in Phi-4 is found in its data processing method rather than the architecture of the model. The Phi-4-14B model is trained with the intention of utilizing the basic causal language modeling:


$$\begin{array}{c} ℒ=-\frac{1}{N}\sum_{i=1}^{N}\sum_{t=1}^{T_{i}}\log P_{\theta }(x_{t}^{\left(i\right)}|x_{<t}^{\left(i\right)}) \end{array}$$ (13)

Here, *N* denotes the serial of the training sequence and $T_{i}$ refers to the length of a single sequence *i*.

The training data collection of Phi-4-14B consists of approximately 1.5 trillion tokens, all these tokens undergoes detailed assessment and filtering. Single record obtains a composite quality score *Q*(*d*), which is calculated as follows:


$$\begin{array}{c} Q(d)=w_{1}q_{\text{edu}}(d)+w_{2}q_{\text{fact}}(d)+w_{3}q_{\text{diverse}}(d)-w_{4}q_{\text{toxic}}(d) \end{array}$$ (14)

In the equation, *q*_edu_ represents educational worth, *q*_fact_ represents factual accuracy, *q*_diverse_ measures lexical diversity, and *q*_toxic_ represents unfavorable or biased content. Individual records that satisfy the threshold condition Q(d)>τ are included in the training dataset.

Furthermore, Phi-4-14B amalgamate high-quality artificial data created by bigger models and verified by human specialists. The procedure is shown as follows:


$$\begin{array}{c} d_{\text{syn}}={\text{LLM}}_{\text{large}}(p_{\text{template}},k_{\text{knowledge}})\to {\text{Human}}_{\text{verify}} \end{array}$$ (15)

Here, *p*_template_ refers to the predefined prompt pattern, and *k*_knowledge_ refers to compiled logical information. Human authentication maintains the logical uniformity and reliability of all the organized data.

The tokenizer used by Phi-4-14B consists of 32,064 tokens, and enables a context length of about 16,384 tokens. The proportions of the feed-forward network are as follows:


$$\begin{array}{c} d_{ff}=4\times d_{h}=20,480 \end{array}$$ (16)

The estimated attribute count is given by the following:


$$\begin{array}{c} N_{\text{params}}=L\times (4d_{h}^{2}+2d_{h}d_{ff})+V\times d_{h}\approx 14\times 10^{9} \end{array}$$ (17)

#### Gemma-2-27B.

Gemma-2 is an advanced open-weights large language model developed by Google DeepMind [48]. The 27-billion-parameter variant was designed to deliver strong performance and inference efficiency through several architectural improvements over the standard Transformer framework.

The model architecture consists of 46 Transformer layers with a hidden dimension of 4608. To improve inference efficiency and reduce memory requirements, Gemma-2-27B employs Grouped-Query Attention (GQA) with 32 attention heads and 16 key-value heads [48]. The attention mechanism can be expressed as:


$$\begin{array}{c} \text{GQA}(Q,K,V)=\text{Concat}({\text{head}}_{1},...,{\text{head}}_{h})W^{O} \end{array}$$ (18)

where multiple query heads share key-value representations, reducing KV-cache memory during autoregressive generation.

Gemma-2 further introduces an interleaved attention mechanism that alternates between global attention and local Sliding Window Attention (SWA) layers. This design enables efficient modeling of both long-range dependencies and local contextual information.

The model employs Rotary Positional Embeddings (RoPE) for positional encoding [48]. In addition, Gemma-2 applies logit soft-capping to prevent excessively large logits during training and inference, thereby improving numerical stability.

Gemma-2-27B was trained on approximately 13 trillion tokens collected from web documents, source code, and scientific articles [48]. The model uses a SentencePiece tokenizer with a vocabulary size of 256,128 tokens, supporting a broad range of languages and scripts. This large multilingual vocabulary enables effective tokenization across diverse linguistic domains and writing systems.

#### Qwen-2.5-72B.

The largest model evaluated in this study is Qwen-2.5-72B-Instruct, an instruction-tuned large language model developed by Alibaba Cloud [49]. The model contains approximately 72 billion parameters and was trained on a large-scale multilingual corpus comprising approximately 18 trillion tokens. Qwen-2.5 supports more than 29 languages and demonstrates strong performance in reasoning, coding, mathematics, and multilingual understanding.

The architecture consists of 80 Transformer layers with a hidden dimension of 8192. To improve inference efficiency and reduce memory consumption, Qwen-2.5-72B employs Grouped-Query Attention (GQA) with 64 query attention heads and 8 key-value heads [44]. The head configuration is given by:


$$\begin{array}{c} h_{\text{query}}=64,\;h_{\text{kv}}=8,\;\text{ratio}=\frac{h_{\text{query}}}{h_{\text{kv}}}=8 \end{array}$$ (19)

The attention computation for a single group can be expressed as:


$$\begin{array}{c} {\text{head}}_{i,g}=\text{Attention}(Q_{i}W_{i}^{Q},\;K_{g}W_{g}^{K},\;V_{g}W_{g}^{V}) \end{array}$$ (20)

where *i* denotes the query head index and *g* identifies the corresponding shared key-value group.

Qwen-2.5 further incorporates Rotary Positional Embeddings (RoPE), SwiGLU activation functions, RMSNorm, and Attention QKV bias to improve representation learning and inference stability. The model supports long-context processing and can handle sequences substantially longer than conventional Transformer-based LLMs.

The model utilizes a byte-level BPE tokenizer with a vocabulary size of 151,936 tokens [49]. This large vocabulary facilitates effective tokenization across diverse languages and writing systems, including Bengali. To accommodate the large parameter count within available hardware constraints, the AWQ (Activation-aware Weight Quantization) version of Qwen-2.5-72B was used in this study while preserving strong inference performance.

### Prompting strategies

To improve methodological transparency, we explicitly describe the prompt design procedure adopted in this study. All prompt instructions used in both zero-shot and few-shot settings were written in English, while the input texts and few-shot examples were presented in Bengali. English instructions were selected because the evaluated open-weight LLMs are primarily instruction-tuned using English prompts, thereby providing a consistent and model-agnostic evaluation framework. We did not vary the language of the instructions. Bengali or bilingual instructions could interact differently with the instruction-tuning data of each model, so our results describe performance with English instructions only (see Limitations).

To analyze the in-context learning competence of the applied Large Language Models (LLMs), two prompting techniques were implemented during the experiment.

**Zero-shot prompting:** For the zero-shot approach, a task instruction prompt is given to the model, for example detect the sentiment or emotion. Additionally, no more samples or context are provided with the prompt. Following this, models utilize their general knowledge initiated completely from the provided prompt to create responses for task completion.
**Few-shot prompting (five-shot):** For the few-shot approach, the model is not provided with any instructions; instead, it is given five examples along with the outputs of the examples. Then the model proceeds to generate responses by deducing the task criteria from the five examples. This enhances the accuracy of models in solving the tasks.

The structure of these techniques adheres to the previous papers on in-context learning [8,12], because previously, after giving the models a few samples, the models performed better and adjusted to the new data more, specifically in the case of low-resource language situations.

To ensure a fair comparison, the same prompt template structure was applied across all evaluated models within each task. For few-shot prompting, five labeled examples were selected from the corresponding training dataset and remained identical across all models. A fixed random seed (seed = 42) was used throughout the experiments to ensure reproducibility. The same five demonstrations were used for every test item, and the full prompts are shown in Figs 2–9. For sentiment analysis the demonstrations contain two positive, two negative and one neutral example. For emotion recognition they contain one example each of joy, sadness, fear, disgust and surprise, and no example of anger. For hate speech detection they contain three hate and two not-hate examples, and for fake news detection two fake and three real examples. Since we evaluated only one demonstration set in one order, the few-shot results tell us how this particular set of demonstrations behaves, not how five-shot prompting behaves in general. We return to this point in the Discussion and Limitations sections. No model-specific prompt engineering, optimization, or calibration was performed. This design ensured that performance differences reflected model capability rather than variations in prompt construction.

**Fig 2:**
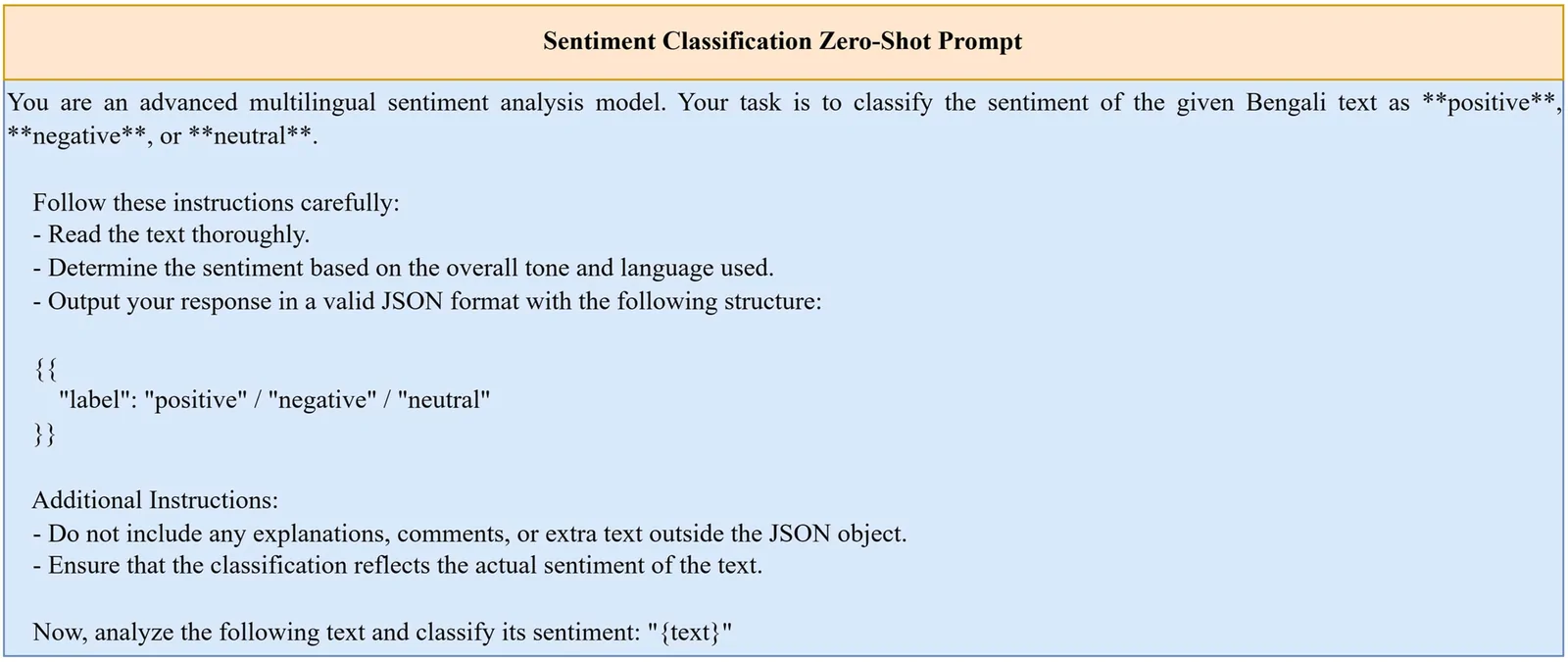
Prompt used for zero-shot Bengali sentiment analysis.

**Fig 3:**
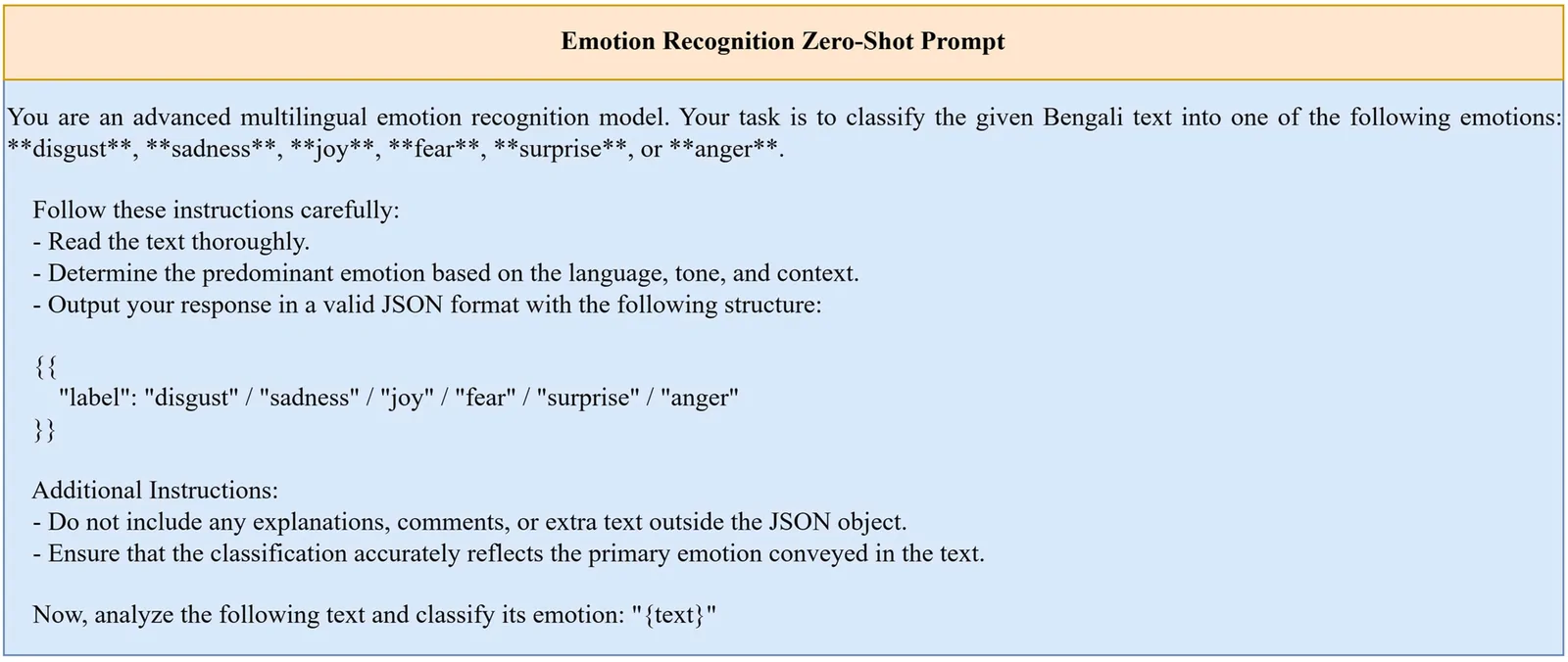
Prompt used for zero-shot Bengali emotion recognition.

**Fig 4:**
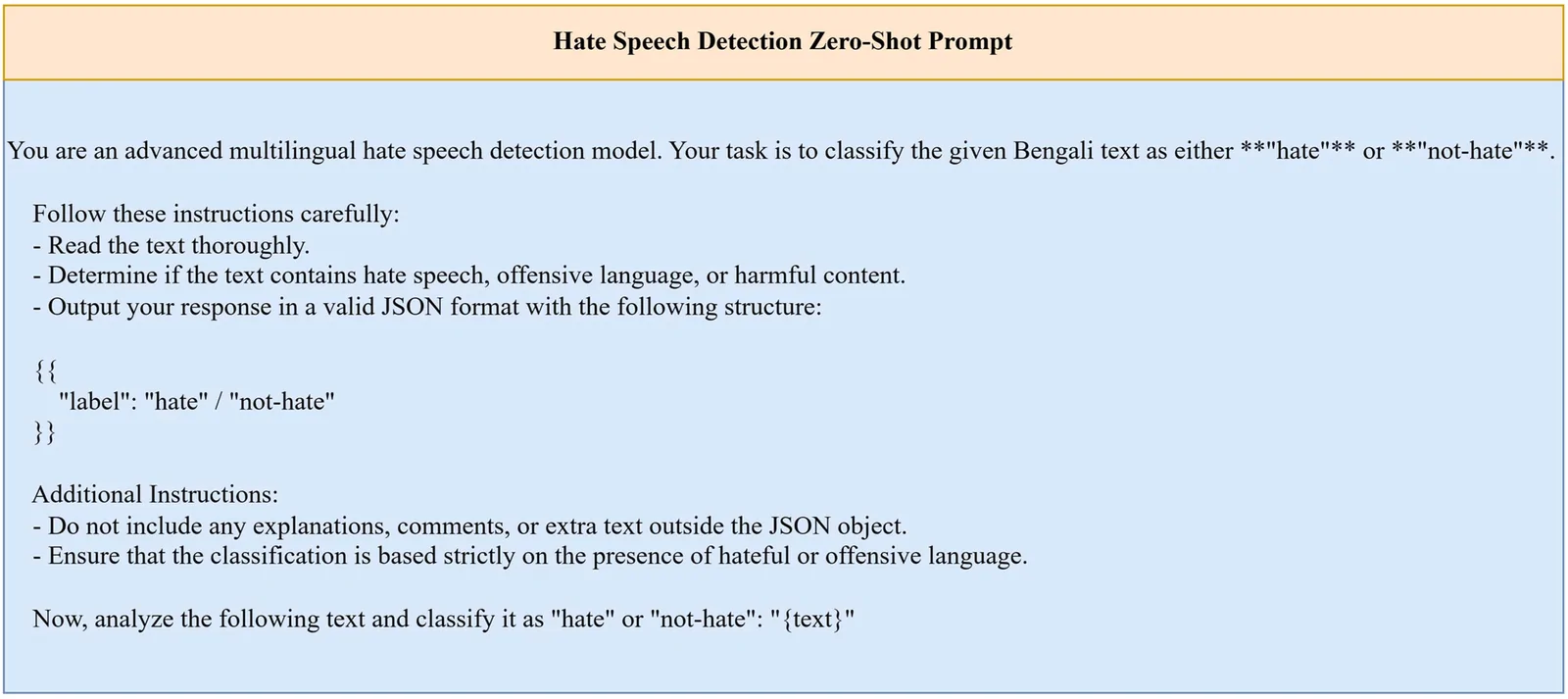
Prompt used for zero-shot Bengali hate speech detection.

**Fig 5:**
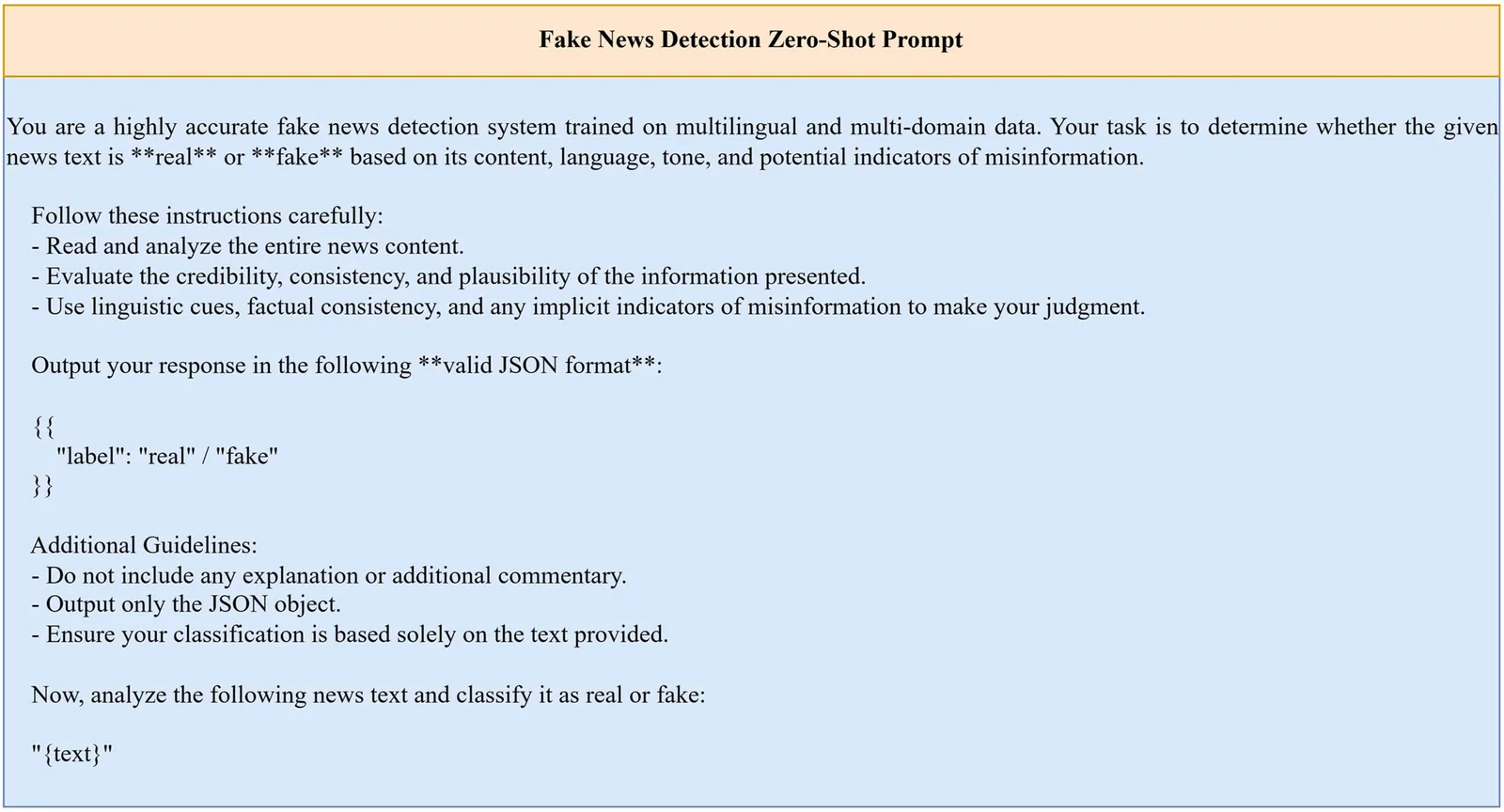
Prompt used for zero-shot Bengali fake news detection.

**Fig 6:**
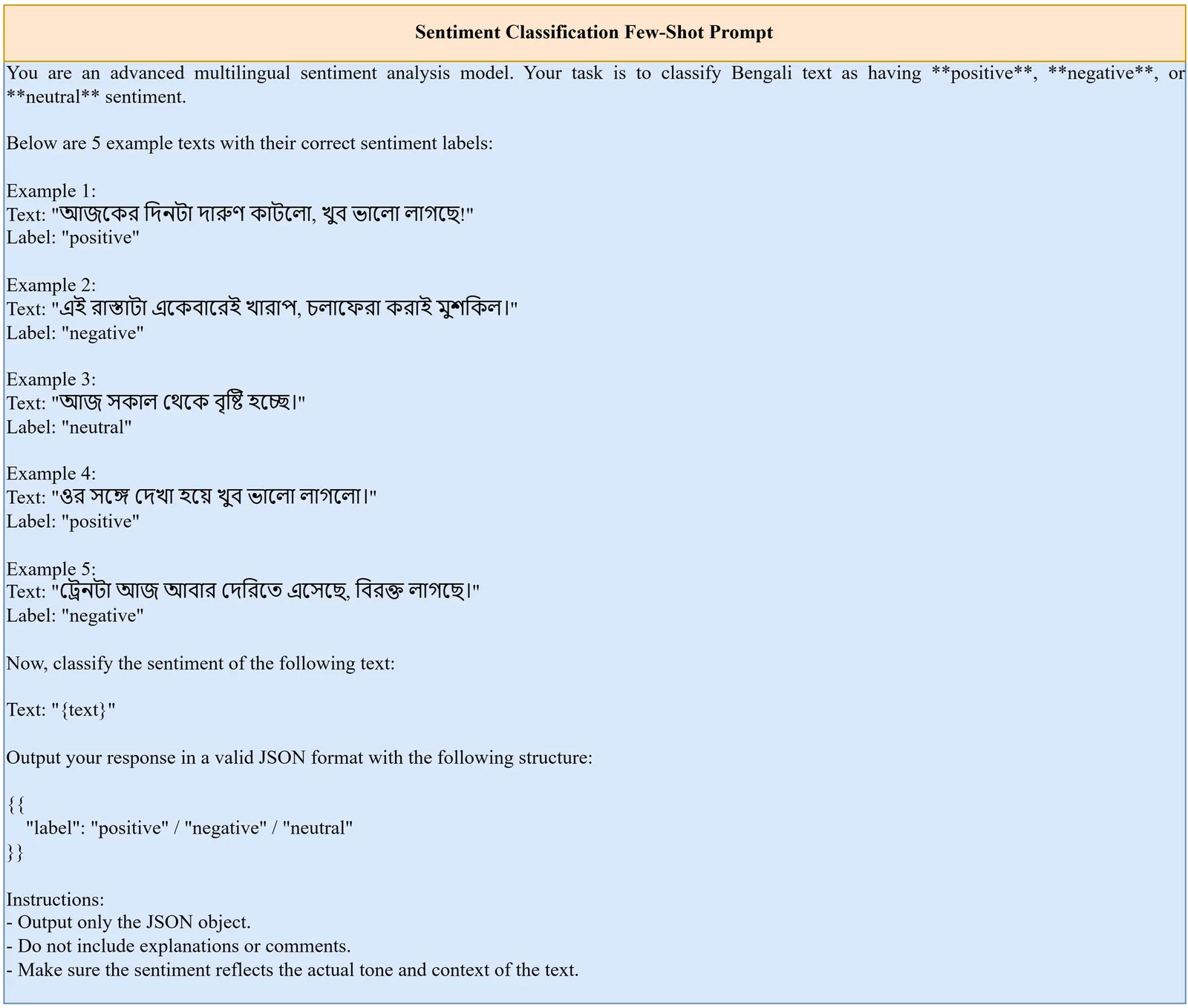
Prompt used for few-shot Bengali sentiment analysis.

**Fig 7:**
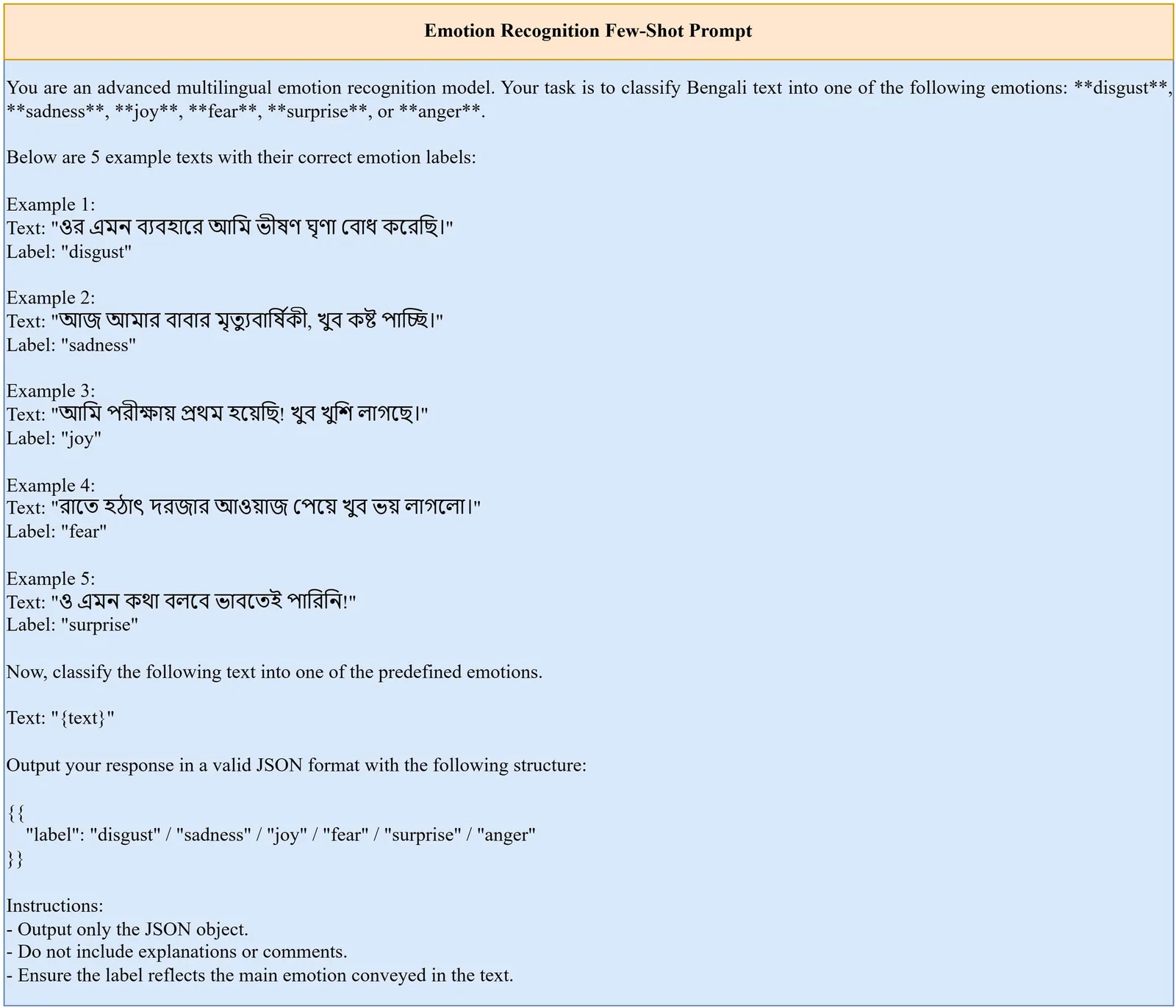
Prompt used for few-shot Bengali emotion recognition.

**Fig 8:**
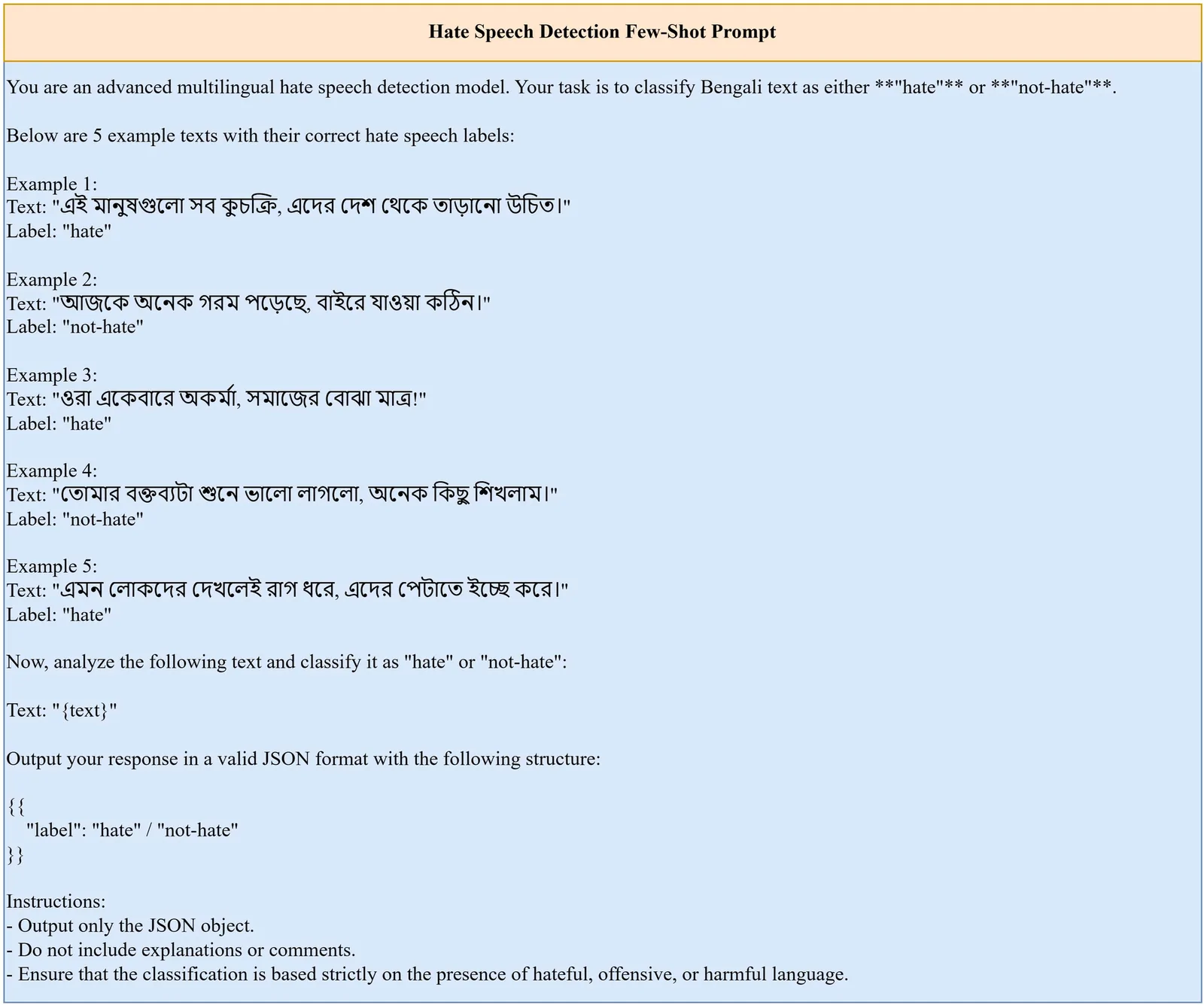
Prompt used for few-shot Bengali hate speech detection.

**Fig 9:**
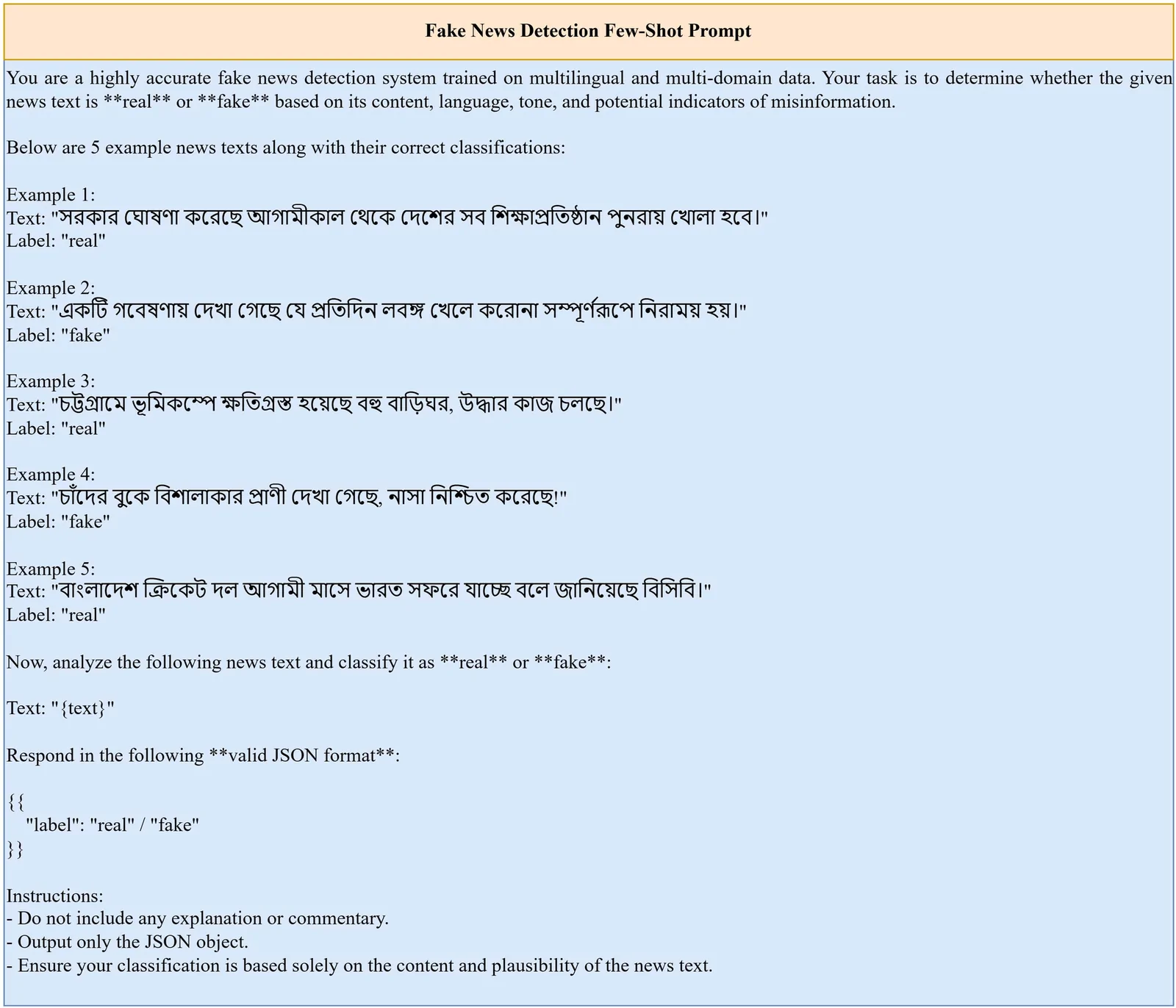
Prompt used for few-shot Bengali fake news detection.

Task-related prompts were generated for Bengali text classification, the four types of classifications include *Sentiment Analysis*, *Emotion Recognition*, *Hate Speech Detection*, and *Fake News Detection*. The prompt was drafted individually to properly describe the task, explain the label space, and stipulate the necessary JSON output pattern for uniform parsing and assessment. The prompts used for the zero-shot setting across these tasks are illustrated in Figs 2–5, respectively. Similarly, the prompts used for the few-shot setting are presented in Figs 6–9.

This consistent pattern enables equal comparison among the LLMs. We differentiate the zero-shot and few-shot systems to analyze if the incorporation of contextual samples enhanced the classification performance and accuracy for Bengali text classification.

### Experimental setup

All model evaluations were conducted using both the *vLLM* inference engine and the Hugging Face Transformers framework. Two computing environments were used during this study. A local workstation equipped with an AMD Ryzen 5 8400F processor (4.20 GHz, 6-core), 16 GB RAM, and an NVIDIA GeForce RTX 4060 GPU (8 GB VRAM) was employed for prompt development, preliminary testing, and evaluation of smaller models.

For large-scale inference involving Qwen-2.5-72B-Instruct-AWQ, a cloud-based NVIDIA RTX PRO 6000 GPU with approximately 96 GB VRAM was utilized through the RunPod cloud platform. The model was deployed in its AWQ-quantized form using AutoAWQ and GPTQModel backends within the Transformers pipeline. This allowed us to improve memory efficiency while maintaining predictive performance. This cloud-based configuration was necessary because the memory requirements of the 72B-parameter model exceed the capacity of an 8 GB consumer-grade GPU. No multi-GPU cluster was used. All Qwen-2.5-72B-Instruct-AWQ inference experiments were conducted on a single cloud-based NVIDIA RTX PRO 6000 GPU through the RunPod platform.

To ensure a fair comparison, all models were evaluated using a unified deterministic decoding configuration, as detailed in Table 5. Generation was performed using greedy decoding (do_sample = False) with a maximum output length of 512 tokens. A fixed random seed of 42 was used throughout the experiments to ensure reproducibility. The same decoding configuration was applied across all datasets and prompting strategies so that performance differences primarily reflected model capability rather than variations in generation settings. We used Python 3.12, PyTorch 2.5.1 (CUDA 11.8 build), Transformers 4.46.3, torchao 0.6.1 and AutoAWQ 0.2.9. Metrics were computed with scikit-learn and the statistical tests with SciPy. Each combination of model, task and prompting setting was run once.

**Table 5:** Inference configuration parameters.

Parameter	Value
Random Seed	42
Decoding Strategy	Greedy Decoding (do_sample = False)
Max Tokens	512
Cloud GPU	NVIDIA RTX PRO 6000 (96 GB VRAM)
Cloud Platform	RunPod
Local Development GPU	NVIDIA GeForce RTX 4060 (8 GB VRAM)
Processor	AMD Ryzen 5 8400F (4.20 GHz, 6-Core)
System RAM	16 GB
Quantization	AWQ (4-bit)
Inference Engine	vLLM, Hugging Face Transformers, AutoAWQ, and GPTQModel
Software	Python 3.12; PyTorch 2.5.1 + cu118; torchao 0.6.1; scikit-learn; SciPy

All experiments were executed on 64-bit operating systems with CUDA and PyTorch properly configured. The combination of vLLM, the Hugging Face Transformers pipeline, AWQ quantization, and cloud-based GPU resources provided through RunPod enabled efficient and reproducible evaluation of all models, including Qwen-2.5-72B-Instruct-AWQ.

### Evaluation metrics

The models were evaluated using established evaluation metrics: *Accuracy*, *Precision*, *Recall*, and *Macro-averaged F1-score*.

Precision and Recall demonstrate the relationship between false positives and false negatives. Given the presence of class imbalance in some of our datasets, the Macro-averaged F1-score was selected as the primary robust metric for evaluation, as recommended for datasets where both classes matter equally. Unlike Accuracy, which may provide misleadingly high values in imbalanced scenarios by favoring majority-class predictions, the Macro-averaged F1-score computes the F1-score independently for each class and then averages them. This ensures that both majority and minority classes contribute equally to the final score, providing a more balanced assessment of classification performance.

While Accuracy provides the overall rate of correct predictions [50], it is reported alongside the Macro-averaged F1-score rather than serving as the sole primary metric due to its limitations in imbalanced classification settings.

One point deserves a note. The test sets of the two binary tasks are exactly balanced (500/500 for hate speech detection and 254/254 for fake news detection). When the classes are balanced, the Macro-F1 and the support-weighted F1 are mathematically the same number, which is why the two averaging schemes give identical values for these two tasks.

Moreover, a *confusion matrix* was generated for each model to obtain True Positives (TP), False Positives (FP), False Negatives (FN), and True Negatives (TN), providing additional insight into model-specific error patterns and class-wise performance.

The formal definitions of the evaluation metrics are as follows:


$$\begin{array}{c} \text{Accuracy}=\frac{TP+TN}{TP+TN+FP+FN} \end{array}$$ (21)

$$\begin{array}{c} {\text{Precision}}_{c}=\frac{TP_{c}}{TP_{c}+FP_{c}} \end{array}$$ (22)

$$\begin{array}{c} {\text{Recall}}_{c}=\frac{TP_{c}}{TP_{c}+FN_{c}} \end{array}$$ (23)

$$\begin{array}{c} {\text{F1-score}}_{c}=\frac{2\times {\text{Precision}}_{c}\times {\text{Recall}}_{c}}{{\text{Precision}}_{c}+{\text{Recall}}_{c}} \end{array}$$ (24)

$$\begin{array}{c} \text{Macro-F1}=\frac{1}{C}\sum_{c=1}^{C}{\text{F1-score}}_{c} \end{array}$$ (25)

Here, *c* denotes an individual class, and *C* represents the total number of classes.

- **Accuracy** calculates the overall performance by taking the ratio of correct predictions to the total number of predictions.
- **Precision** measures the proportion of correctly predicted positive instances among all positive predictions made by the model.
- **Recall** assesses the proportion of actual positive instances that were correctly identified by the model.
- **Macro F1-score** computes the F1-score for each label and finds their unweighted mean. This treats all classes equally, making it highly effective for evaluating imbalanced datasets where minority classes are as important as majority classes.

### Statistical significance testing

To assess whether the observed performance differences between the evaluated Large Language Models (LLMs) were statistically meaningful, we performed paired permutation tests on the model predictions. Because all models were evaluated on the same test instances, a paired testing framework was adopted to account for the dependency between predictions and to provide a more reliable comparison.

For each dataset and prompting setting, the observed differences in Accuracy and Macro F1-score between the compared models were first calculated. A permutation-based resampling procedure was then applied by randomly exchanging the predictions of the two models for individual test samples while preserving the ground-truth labels. This process was repeated 1,000 times to generate a null distribution representing the expected performance differences under the assumption that both models performed equivalently.

The p-value was estimated as the proportion of permutations that produced an absolute performance difference greater than or equal to the observed absolute difference (i.e., a two-sided test); when none of the 1,000 permutations reaches the observed difference, the p-value is reported as <0.001 and taken as 0.001 when correcting for multiple comparisons. Statistical significance was assessed using a significance level of α=0.05. A p-value below 0.05 indicates that the observed difference is unlikely to have occurred by chance and can therefore be considered statistically significant.

A permutation-based approach was selected because it is non-parametric and does not require assumptions regarding the underlying distribution of evaluation metrics. Consequently, it is well suited for comparing classification models on shared benchmark datasets. In this study, the test was applied to compare the prediction outputs of Qwen-2.5-72B and Gemma-2-27B, the two strongest-performing models across the evaluated tasks; it therefore concerns only this single pair of models.

**Multi-model paired analysis and multiple-comparison correction.** A test between the two strongest models says nothing about the other four, so we also analysed all six models together on the same test items. For each dataset and prompting setting we first applied Cochran’s Q test [51], which tests whether the six models have the same accuracy (*k* = 6, df = 5). We then compared all 15 pairs of models with the exact (binomial) McNemar test [52,53], which uses only the items on which the two models disagree, and corrected the 15 p-values within each dataset and setting with the Holm–Bonferroni step-down procedure [54,55]. The same McNemar test was used to compare the zero-shot and few-shot predictions of each model on the same items; these 24 p-values were Holm-corrected as one family. Finally, the 16 p-values from the Qwen-2.5-72B versus Gemma-2-27B permutation tests were also Holm-corrected as one family.

### Confidence interval estimation

To quantify the uncertainty associated with model performance estimates, 95% confidence intervals (CIs) were computed for the Macro F1-score of all six models using bootstrap resampling. For each model and evaluation setting, 1,000 bootstrap samples were generated by randomly sampling the test instances with replacement. The Macro F1-score was calculated for each bootstrap sample, and the 2.5th and 97.5th percentiles of the resulting distribution were used to construct the 95% confidence interval. This non-parametric approach does not assume any specific distribution of the evaluation metric and is widely used for estimating uncertainty in machine learning evaluation studies.

## Results and analysis

This section presents the performance of various LLMs on two prompting settings across four classification tasks. The associated results for each prompting strategy are described as follows.

### Statistical significance analysis

To determine whether the observed performance differences between the evaluated LLMs were statistically significant, paired permutation tests were conducted using 1,000 random permutations. The analysis focused on the two strongest-performing models, Qwen-2.5-72B and Gemma-2-27B, across all datasets and prompting settings. Table 6 presents the corresponding p-values for accuracy and macro F1-score.

**Table 6:** Paired permutation test results comparing Qwen-2.5-72B and Gemma-2-27B, with Holm-adjusted p-values (family of 16 tests).

Dataset	Setting	Acc. p (raw)	Acc. p (Holm)	Macro-F1 p (raw)	Macro-F1 p (Holm)
Emotion	Zero-shot	1.000	1.000	0.943	1.000
Emotion	Few-shot	0.003	0.039	0.016	0.160
Fake News	Zero-shot	0.475	1.000	0.306	1.000
Fake News	Few-shot	0.876	1.000	0.658	1.000
Hate Speech	Zero-shot	0.007	0.077	0.004	0.048
Hate Speech	Few-shot	0.001	0.016	<0.001	0.016
Sentiment	Zero-shot	<0.001	0.016	0.101	0.707
Sentiment	Few-shot	0.023	0.207	0.055	0.440

The results reveal that the statistical significance of the observed performance differences varies across tasks. Before correction, the differences for hate speech detection are significant in both settings and for both metrics, and so are the differences for emotion recognition under few-shot prompting. After Holm correction over the 16 tests, five of them survive: hate speech detection under few-shot prompting for both accuracy and Macro-F1 (adjusted *p* = 0.016), hate speech detection under zero-shot prompting for Macro-F1 only (adjusted *p* = 0.048; accuracy *p* = 0.077), emotion recognition under few-shot prompting for accuracy only (adjusted *p* = 0.039; Macro-F1 *p* = 0.160), and sentiment analysis under zero-shot prompting for accuracy only (adjusted *p* = 0.016; Macro-F1 *p* = 0.707).

In contrast, no statistically significant differences were observed for fake-news detection in either prompting setting (*p* > 0.05), suggesting that Qwen-2.5-72B and Gemma-2-27B achieved comparable performance on this task. For sentiment analysis, the findings were mixed. After correction, the accuracy difference is significant only under zero-shot prompting, where Gemma-2-27B is ahead, and the Macro-F1 differences are not significant in either setting. Note that these tests involve only Qwen-2.5-72B and Gemma-2-27B; the other models are compared in the multi-model analysis below.

Overall, these results suggest that numerical differences in performance metrics should be interpreted with caution, as they do not always correspond to statistically significant performance gains. Therefore, both evaluation metrics and statistical significance tests should be considered when comparing LLM performance.

### Multi-model paired analysis

Since the permutation tests above compare only two models, we also tested all six models together. For each dataset and prompting setting, Cochran’s Q test checks whether the six models make correct predictions at the same rate on the same test items. Table 7 shows the results. In every case the test rejects the idea that the models perform equally (*p* < 0.001), which is not surprising given the large performance gaps between the models. The more useful part of the table is the last column, which lists the pairs of models that could not be separated after Holm correction. Three patterns stand out. First, the three smallest models (LLaMA-3.2-3B, Mistral-V3-7B and DeepSeek-R1-8B) often perform at the same level; they cannot be separated on few-shot emotion recognition, few-shot hate speech detection, or zero-shot sentiment analysis. Second, Phi-4-14B is not significantly different from Gemma-2-27B and Qwen-2.5-72B on zero-shot emotion recognition and on few-shot fake news detection. Third, the two largest models cannot be separated on zero-shot emotion recognition, on fake news detection in either setting, or on few-shot sentiment analysis. Where they do differ, the pairwise results agree with the permutation tests in Table 6: Qwen-2.5-72B is more accurate on hate speech detection in both settings (adjusted *p* = 0.020 and 0.005) and on few-shot emotion recognition (*p* = 0.022), while Gemma-2-27B is more accurate on zero-shot sentiment analysis (*p* = 0.001). It is also worth noting that LLaMA-3.2-3B, the smallest model, is never significantly worse than the 8B DeepSeek model, and is actually better on few-shot sentiment analysis (*p* < 0.001). On zero-shot fake news detection it performs the same as the 14B Phi-4 model (77.76% versus 77.36%, *p* = 0.96). Model size alone therefore does not explain the ranking.

**Table 7:** Multi-model paired analysis: Cochran’s Q omnibus test over the six models and Holm-corrected pairwise exact McNemar tests (15 pairs per row). The last column lists the pairs whose accuracies do not differ significantly at α=0.05 after correction.

Dataset	Setting	Cochran’s Q (df = 5)	p	Sig. pairs (Holm)	Pairs not significantly different
Sentiment	Zero-shot	133.1	<0.001	10/15	LLaMA–Mistral; LLaMA–DeepSeek; Mistral–DeepSeek; Phi-4–Gemma; Phi-4–Qwen
Sentiment	Few-shot	193.5	<0.001	10/15	LLaMA–Phi-4; LLaMA–Qwen; Mistral–DeepSeek; Phi-4–Qwen; Gemma–Qwen
Emotion	Zero-shot	263.7	<0.001	11/15	LLaMA–DeepSeek; Phi-4–Gemma; Phi-4–Qwen; Gemma–Qwen
Emotion	Few-shot	130.9	<0.001	12/15	LLaMA–Mistral; LLaMA–DeepSeek; Mistral–DeepSeek
Hate Speech	Zero-shot	282.7	<0.001	14/15	LLaMA–DeepSeek
Hate Speech	Few-shot	159.8	<0.001	12/15	LLaMA–Mistral; LLaMA–DeepSeek; Mistral–DeepSeek
Fake News	Zero-shot	140.1	<0.001	11/15	LLaMA–DeepSeek; LLaMA–Phi-4; DeepSeek–Phi-4; Gemma–Qwen
Fake News	Few-shot	202.5	<0.001	10/15	LLaMA–DeepSeek; Mistral–DeepSeek; Phi-4–Gemma; Phi-4–Qwen; Gemma–Qwen

Table 8 asks a different question: for each model and task, did adding the five demonstrations actually change the predictions? Because the zero-shot and few-shot runs use the same test items, we again used the exact McNemar test, with Holm correction over the 24 comparisons. Only four of them are significant. Three are improvements, and all involve the two smallest models: LLaMA-3.2-3B gains 4.9 points on sentiment analysis and 7.3 points on hate speech detection, and Mistral-V3-7B gains 9.3 points on emotion recognition. One is a clear loss: LLaMA-3.2-3B drops 8.5 points on fake news detection. For Phi-4-14B, Gemma-2-27B and Qwen-2.5-72B, none of the twelve changes reaches significance. Even the largest change for Qwen-2.5-72B, + 4.0 points on emotion recognition, has an adjusted *p* of 0.18, and its 1.5-point drop on hate speech detection has *p* = 0.98. These results should be kept in mind when reading the few-shot scores below. They come from a single fixed set of demonstrations, and for most model–task combinations the difference between the two settings is no larger than what item-level variation alone would produce.

**Table 8:** Effect of five-shot prompting relative to zero-shot prompting for each model and task: Change in accuracy in percentage points and Holm-adjusted p-value of the exact McNemar test on the paired predictions (24 tests). * indicates significance at α=0.05 after correction.

Model	Sentiment	Emotion	Hate Speech	Fake News
LLaMA-3.2-3B	+4.9 (0.009)*	+0.5 (1.000)	+7.3 (0.015)*	−8.5 (0.007)*
Mistral-V3-7B	−3.4 (0.074)	+9.3 (0.001)*	−1.6 (1.000)	+2.0 (1.000)
DeepSeek-R1-8B	−0.2 (1.000)	+0.6 (1.000)	+2.6 (1.000)	−5.1 (0.751)
Phi-4-14B	−1.5 (0.538)	−3.8 (0.100)	−1.5 (1.000)	+4.3 (0.052)
Gemma-2-27B	+0.5 (1.000)	−0.6 (1.000)	−2.2 (0.192)	+0.0 (1.000)
Qwen-2.5-72B	+2.2 (0.188)	+4.0 (0.179)	−1.5 (0.980)	+0.8 (1.000)

### Confidence interval analysis

To account for the varying test-set sizes across the evaluated tasks, 95% bootstrap confidence intervals were computed for the Macro F1-score of all six models. Table 9 reports the corresponding confidence intervals.

**Table 9:** 95% bootstrap confidence intervals for Macro F1-score of all six models (1,000 resamples; values for Qwen-2.5-72B and Gemma-2-27B are unchanged from the previous analysis).

Dataset (Test Size)	Prompt Setting	LLaMA-3.2-3B	Mistral-V3-7B	DeepSeek-R1-8B	Phi-4-14B	Gemma-2-27B	Qwen-2.5-72B
Sentiment (1586)	Zero-shot	49.62 (47.17, 52.03)	50.20 (47.73, 52.66)	49.90 (47.38, 52.28)	57.73 (55.26, 60.06)	58.66 (56.38, 61.25)	56.84 (54.45, 59.22)
Sentiment (1586)	Few-shot	54.61 (52.31, 57.06)	48.09 (45.68, 50.56)	49.47 (47.13, 51.82)	56.44 (53.98, 58.81)	60.68 (58.37, 63.18)	58.82 (56.45, 61.11)
Emotion (625)	Zero-shot	45.88 (41.90, 49.79)	36.08 (32.19, 39.88)	44.08 (40.07, 47.73)	59.89 (56.28, 63.28)	61.80 (57.95, 65.39)	61.91 (58.08, 65.96)
Emotion (625)	Few-shot	46.35 (41.84, 50.22)	45.18 (40.83, 49.18)	45.85 (42.40, 49.47)	57.90 (54.23, 61.03)	63.01 (59.59, 66.37)	66.80 (63.11, 70.17)
Hate Speech (1000)	Zero-shot	54.22 (51.01, 57.60)	64.19 (61.03, 67.09)	56.40 (53.60, 59.61)	72.17 (69.50, 74.90)	76.11 (73.38, 78.71)	79.88 (77.49, 82.28)
Hate Speech (1000)	Few-shot	62.85 (59.79, 65.74)	63.07 (59.89, 66.10)	61.47 (58.45, 64.52)	70.56 (67.69, 73.32)	73.39 (70.56, 76.04)	78.25 (75.65, 80.67)
Fake News (508)	Zero-shot	77.72 (74.21, 81.10)	55.45 (50.79, 59.82)	72.39 (68.50, 76.47)	76.91 (73.09, 80.57)	84.33 (80.87, 87.37)	82.91 (79.57, 86.11)
Fake News (508)	Few-shot	66.27 (61.54, 70.49)	58.93 (54.67, 63.25)	65.59 (61.31, 69.80)	81.48 (77.63, 84.80)	84.17 (80.98, 87.34)	83.68 (80.28, 86.80)

As expected, datasets with smaller test sizes, such as Emotion Recognition and Fake News Detection, exhibited relatively wider confidence intervals than larger datasets. In contrast, the Sentiment Analysis dataset, which contains the largest test set, produced comparatively narrower intervals. The intervals are between 4.7 and 9.0 Macro-F1 points wide, and those of neighbouring models often overlap. For example, Phi-4-14B, Gemma-2-27B and Qwen-2.5-72B overlap on zero-shot emotion recognition, and Gemma-2-27B and Qwen-2.5-72B overlap on fake news detection in both settings. This agrees with the pairwise tests above: a difference of a few points between two neighbouring models should not be read as a real difference.

### Zero-shot performance

Table 10 presents the zero-shot performance of all evaluated large language models (LLMs) across the four Bengali text classification tasks. For the sentiment Analysis task, Gemma-2-27B reported the overall best results on accuracy (62.04%), recall (62.04%), and Macro-F1 score (58.66%), generalizing well even without in-context examples. In comparison, Qwen-2.5-72B achieved the best precision with 66.12%, meaning it was better at suppressing false positives and generating confident predictions.

**Table 10:** Zero-shot evaluation results of large language models (LLMs) on various Bengali text classification tasks.

Model	Sentiment Analysis				Emotion Recognition			
	Acc.	P.	R.	Macro-F1	Acc.	P.	R.	Macro-F1
LLaMA-3.2-3B	52.21	54.62	52.21	49.62	46.24	49.08	46.24	45.88
Mistral-V3-7B	51.32	54.49	51.32	50.20	36.96	49.30	36.96	36.08
DeepSeek-R1-8B	50.82	55.15	50.82	49.90	45.92	47.48	45.92	44.08
Phi-4-14B	59.84	64.11	59.84	57.73	59.84	65.48	59.84	59.89
Gemma-2-27B	62.04	65.12	62.04	58.66	61.76	68.47	61.76	61.80
Qwen-2.5-72B	58.07	**66.12**	58.07	56.84	61.76	67.67	61.76	61.91
**Model**	**Hate Speech Detection**				**Fake News Detection**			
	**Acc.**	**P.**	**R.**	**Macro-F1**	**Acc.**	**P.**	**R.**	**Macro-F1**
LLaMA-3.2-3B	57.10	59.49	57.10	54.22	77.76	77.95	77.76	77.72
Mistral-V3-7B	64.70	65.58	64.70	64.19	61.61	76.02	61.61	55.45
DeepSeek-R1-8B	59.70	63.91	59.70	56.40	72.44	72.61	72.44	72.39
Phi-4-14B	72.50	73.62	72.50	72.17	77.36	79.68	77.36	76.91
Gemma-2-27B	76.50	78.33	76.50	76.11	84.45	85.57	84.45	84.33
Qwen-2.5-72B	79.90	80.05	79.90	79.88	83.27	86.26	83.27	82.91

For the Emotion Recognition benchmark, Qwen-2.5-72B and Gemma-2-27B exhibited comparable results, with an accuracy of 61.76%. Their Macro-F1 scores of 61.91% and 61.80%, respectively, indicate a moderate level of emotion classification performance without task-specific fine-tuning.

Among the Hate Speech Detection systems, Qwen-2.5-72B achieved the best results in terms of overall performance, with an accuracy of 79.90% and a Macro-F1 score of 79.88%, the strongest hate speech classification performance among the evaluated models. We achieved the best accuracy of 84.45% and a Macro-F1 score of 84.33% in Fake News Detection on Gemma-2-27B, the best fake news classification performance in this setting.

Overall, the two largest models, Qwen-2.5-72B and Gemma-2-27B, obtain the highest scores on every task. The ranking does not simply follow model size, however. Gemma-2-27B beats the 72B Qwen model on sentiment analysis and fake news detection, and the 3B LLaMA model is on par with the 14B Phi-4 model on zero-shot fake news detection (77.72% versus 76.91% Macro-F1; Holm-adjusted McNemar *p* = 0.96). Because each model size comes from a different model family, with its own pre-training corpus, tokenizer and instruction tuning, we describe the advantage of the largest models as an association with scale and multilingual pre-training rather than as an effect of parameter count. Smaller models such as LLaMA-3.2-3B, Mistral-V3-7B, and DeepSeek-R1-8B were less competitive. Their smaller capacity and their more limited exposure to Bengali during pre-training are both plausible reasons, but our design cannot tell the two apart.

### Few-shot performance

Table 11 presents the performance of the evaluated large language models (LLMs) in the few-shot setting, where each model was provided with five labelled examples per task. Overall, Qwen-2.5-72B performs the best in the Emotion Recognition and Hate Speech Detection tasks, with a Macro-F1 score of 66.80% and 78.25%, respectively. Gemma-2-27B achieves outstanding results in the Sentiment Analysis and Fake News Detection tasks with a Macro-F1 score of 60.68% and 84.17%. Qwen-2.5-72B and Gemma-2-27B therefore remain the strongest models under few-shot prompting. At the same time, neither of them changes significantly compared with its own zero-shot predictions (Table 8).

**Table 11:** Few-shot evaluation results of large language models (LLMs) on various Bengali text classification tasks.

Model	Sentiment Analysis				Emotion Recognition			
	Acc.	P.	R.	Macro-F1	Acc.	P.	R.	Macro-F1
LLaMA-3.2-3B	57.12	57.50	57.12	54.61	46.72	63.35	46.72	46.35
Mistral-V3-7B	47.92	58.03	47.92	48.09	46.24	57.79	46.24	45.18
DeepSeek-R1-8B	50.63	54.20	50.63	49.47	46.56	45.69	46.56	45.85
Phi-4-14B	58.32	64.24	58.32	56.44	56.00	64.95	56.00	57.90
Gemma-2-27B	62.55	67.93	62.55	60.68	61.12	71.66	61.12	63.01
Qwen-2.5-72B	60.28	66.86	60.28	58.82	65.76	70.79	65.76	66.80
**Model**	**Hate Speech Detection**				**Fake News Detection**			
	**Acc.**	**P.**	**R.**	**Macro-F1**	**Acc.**	**P.**	**R.**	**Macro-F1**
LLaMA-3.2-3B	64.40	67.28	64.40	62.85	69.29	80.05	69.29	66.27
Mistral-V3-7B	63.10	63.14	63.10	63.07	63.58	74.84	63.58	58.93
DeepSeek-R1-8B	62.30	63.46	62.30	61.47	67.32	71.69	67.32	65.59
Phi-4-14B	71.00	72.33	71.00	70.56	81.69	83.25	81.69	81.48
Gemma-2-27B	74.30	78.15	74.30	73.39	84.45	87.03	84.45	**84.17**
Qwen-2.5-72B	78.40	79.18	78.40	**78.25**	84.06	87.50	84.06	83.68

Compared with the zero-shot setting, the effect of few-shot prompting is task-dependent and varies across models. Five of the six models score a higher Macro-F1 on Emotion Recognition with demonstrations, but only the gain of Mistral-V3-7B (from 36.08% to 45.18%) is significant after Holm correction. The gains of Qwen-2.5-72B (61.91% to 66.80%) and Gemma-2-27B (61.80% to 63.01%) are not (Table 8). However, the benefits are not universal. For some tasks, such as Fake News Detection, performance remains largely unchanged or decreases slightly for certain models. Similarly, Qwen-2.5-72B records a small reduction in Hate Speech Detection Macro-F1 score (79.88% to 78.25%) compared with its zero-shot performance. With the single demonstration set used here, in-context examples give the strongest models no measurable benefit, but they do change how the models decide. In hate speech detection, the demonstrations (three hate, two not-hate) made every model label more items as hate: for Gemma-2-27B the share of items predicted as hate rose from 62.7% to 68.5%, and for LLaMA-3.2-3B from 24.9% to 70.4%. This raises recall for the hate class but adds false positives on this balanced test set.

Both Mistral-V3-7B and DeepSeek-R1-8B show relatively weak performance compared with the other models. For example, their Macro-F1 scores in Sentiment Analysis are 48.09% and 49.47%, while in Emotion Recognition they achieve 45.18% and 45.85%. This inferior performance can be explained partly because of the smaller parameter size and less multilingual training, as well as lesser exposure to Bengali in the pre-training. Larger multilingual models such as Qwen-2.5-72B and Gemma-2-27B exhibit more robust classification performance. This is consistent with an advantage of scale and multilingual pre-training, although it does not prove one.

### Class-wise performance analysis

This section discusses the LLMs’ performance across different classes of the four classification tasks. All values in this section and in Figs 10–13 are per-class F1-scores. The Macro-F1 in Tables 10 and 11 is simply the average of these per-class values, so the figures break the Macro-F1 down by class rather than showing a different metric.

**Fig 10:**
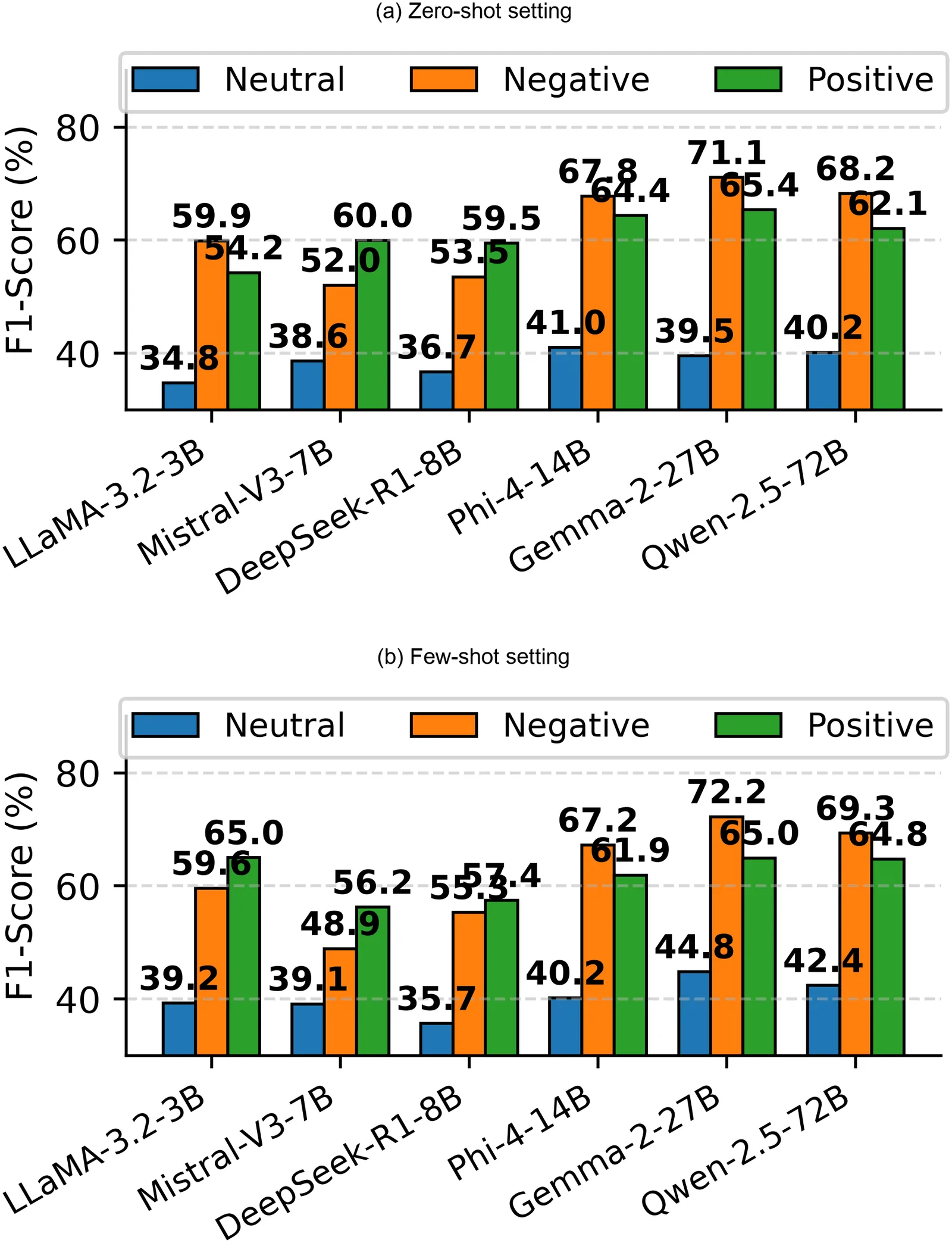
Class-wise sentiment classification performance for two prompt settings across LLMs. Bars show per-class F1-scores (%); their average is the Macro-F1 reported in Tables 10 and 11. (a) Zero-shot setting. (b) Few-shot setting.

**Fig 11:**
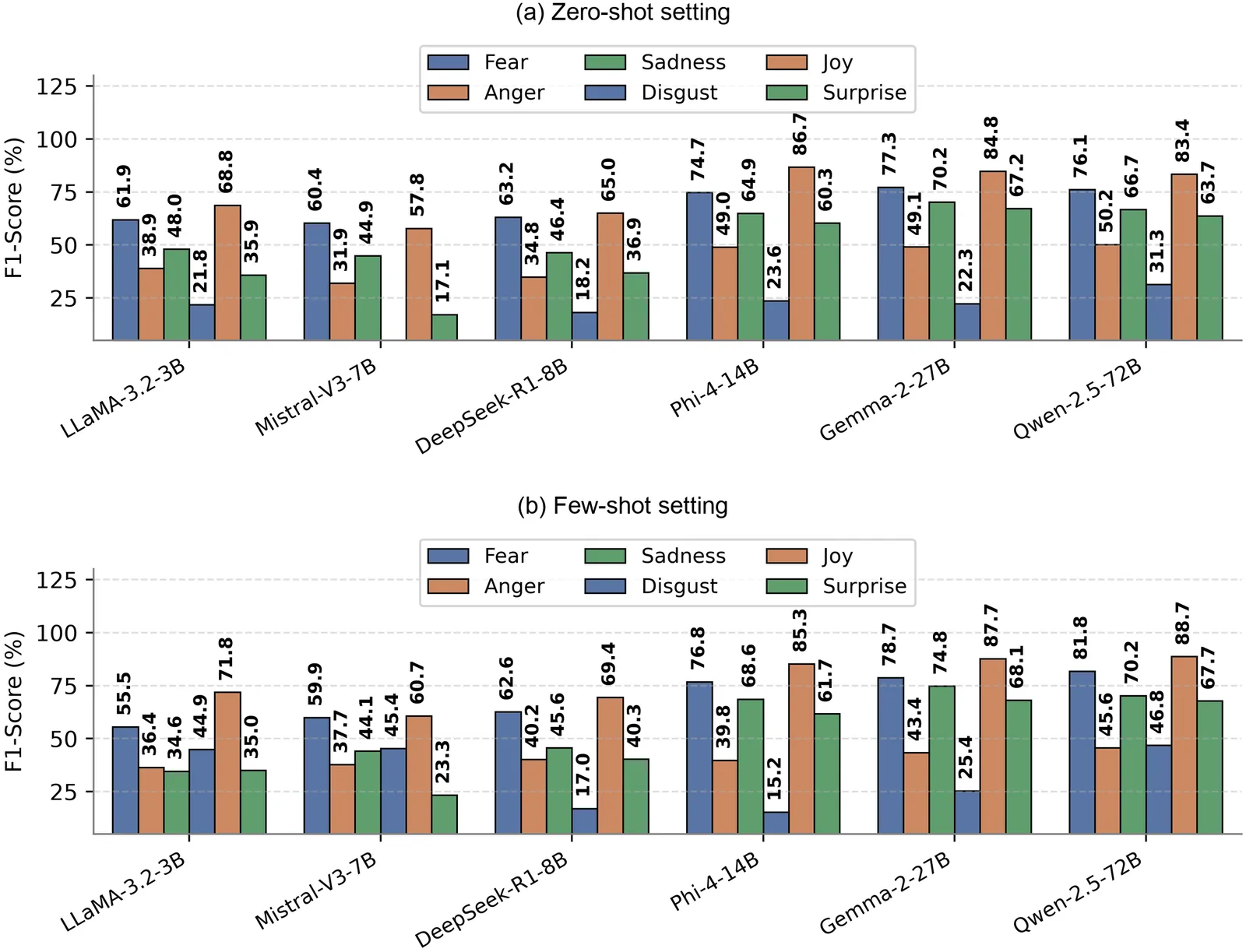
Class-wise emotion recognition performance for two prompt settings across LLMs. Bars show per-class F1-scores (%); their average is the Macro-F1 reported in Tables 10 and 11. (a) Zero-shot setting. (b) Few-shot setting.

**Fig 12:**
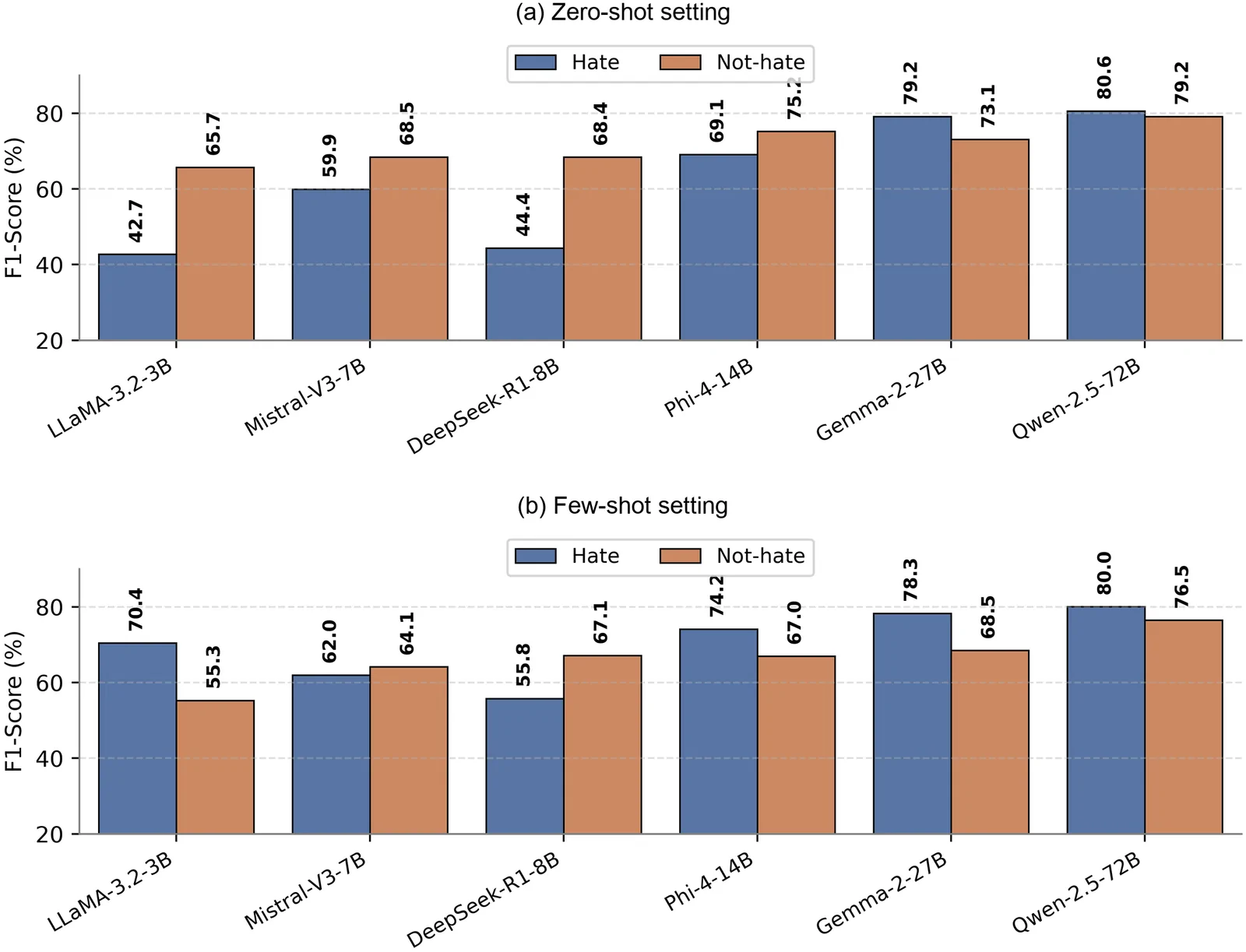
Class-wise hate speech detection performance for two prompt settings across LLMs. Bars show per-class F1-scores (%); their average is the Macro-F1 reported in Tables 10 and 11. (a) Zero-shot setting. (b) Few-shot setting.

**Fig 13:**
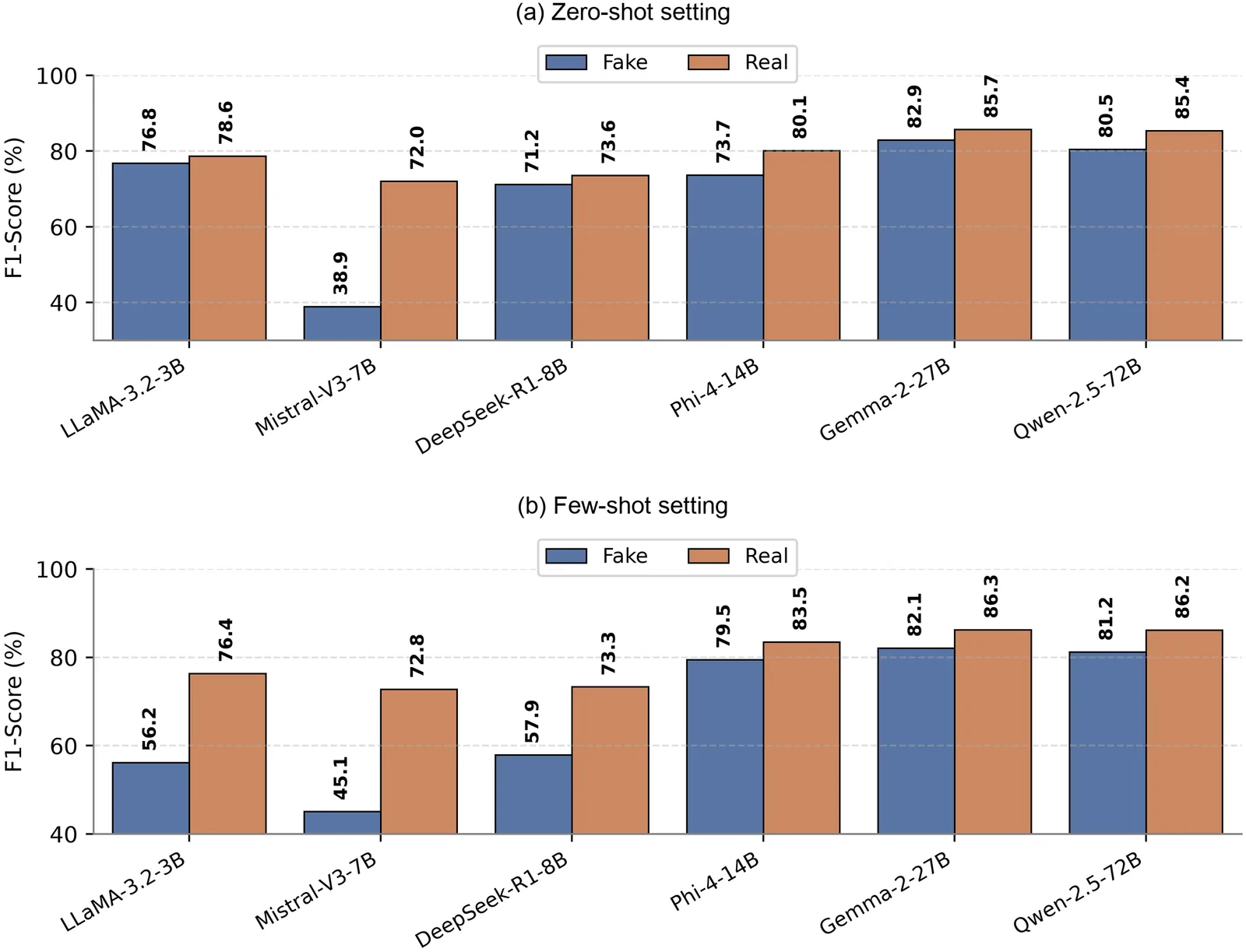
Class-wise fake news detection performance for two prompt settings across LLMs. Bars show per-class F1-scores (%); their average is the Macro-F1 reported in Tables 10 and 11. (a) Zero-shot setting. (b) Few-shot setting.

#### LLMs performance across sentiment categories.

Fig 10 presents the F1-scores of each sentiment class across all large language models (LLMs) for both zero-shot and few-shot prompting settings. In the zero-shot condition, all models achieve higher F1 scores on the negative and positive sentiment classes when comparing with Gemma-2-27B model and Qwen-2.5-72B with the most optimal performance. However, the neutral class remains inferior in F1-score across all models, consistently showing that separating neutral sentiment is still difficult in the absence of context examples. This difficulty is partially exacerbated by the fact that the neutral class is underrepresented in the dataset (361 samples compared to 654 positive and 571 negative).

The trend is similar in the few-shot scenario, with substantial improvements for the neutral class observed across several models (including DeepSeek-R1-8B, which struggles with neutral sentiment under zero-shot conditions but benefits from few-shot examples). For example, Gemma-2-27B shows an increase of approximately five percentage points in the neutral category when moving from zero-shot to few-shot prompting. So for several models the demonstrations improve the neutral class and give a more even per-class profile. Whether this means the models handle ambiguous sentiment better, or simply predict neutral more often, cannot be decided from these figures alone (see the error analysis).

#### LLMs performance across emotion categories.

Fig 11 shows the F1 scores across emotion categories. As we can see in the zero-shot setting, the performance of LLMs is highly heterogeneous on emotion types, from Joy and Fear classes with higher F1 scores to Disgust and Anger categories being more difficult to classify accurately. Qwen-2.5-72B and Gemma-2-27B again show strong performance, except in negative emotions such as Anger and Disgust. While few-shot prompting with Qwen-2.5-72B improves performance by 15% in the Disgust class compared to zero-shot prompting, it results in a 5% decrease in the Anger class. This trade-off suggests a potential semantic overlap between expressions of anger and disgust in the dataset, leading the model to occasionally misclassify nuanced emotional cues. For Qwen-2.5-72B, Joy and Fear improve by more than five points (83.4% to 88.7% and 76.1% to 81.8%), while Sadness and Surprise improve by 3.5 and 4.0 points (66.7% to 70.2% and 63.7% to 67.7%). It is worth noting that the few-shot prompt contains no anger example, and that the Anger F1 fell under few-shot prompting for four of the six models, including the three strongest (Phi-4-14B from 49.0% to 39.8%, Gemma-2-27B from 49.1% to 43.4%, Qwen-2.5-72B from 50.2% to 45.6%). We also notice that the LLaMA-3.2-3B, Mistral-V3-7B, and DeepSeek-R1-8B perform poorly across most of the categories, especially in Anger, Disgust, and Surprise, with both prompting approaches.

#### LLMs performance across hate and not-hate categories.

Fig 12 presents the class-wise F1-scores for the Hate Speech Detection task under both zero-shot and few-shot prompting settings. In the zero-shot setting, most models, including LLaMA-3.2-3B, struggle to identify the hate class accurately. The results show that several smaller or less multilingual models obtain an F1-score below 50% for the hate class, while their performance on the not-hate class remains disproportionately high. This pattern suggests that these models tend to prefer safer non-hateful predictions when no labelled examples are provided. Such imbalance indicates that smaller models may have limited ability to capture socially sensitive and context-dependent Bengali expressions. Similar challenges have been observed in other low-resource languages, where limited representation in pretraining data restricts a model’s ability to grasp culturally grounded expressions.

Conversely, larger models handle the zero-shot setting more effectively. Under zero-shot prompting, Qwen-2.5-72B achieves an F1-score of 80.6% for the hate class and 79.2% for the not-hate class. Similarly, Gemma-2-27B records 79.2% for the hate class and 73.1% for the not-hate class.

In the few-shot setting, LLaMA-3.2-3B improves clearly: its hate-class F1 rises from 42.7% to 70.4% and its Macro-F1 from 54.22% to 62.85%, the only significant few-shot change on this task (Table 8). Phi-4-14B gains on the hate class (69.1% to 74.2%) but loses on the not-hate class (75.2% to 67.0%), so its Macro-F1 drops slightly (72.17% to 70.56%). What all models have in common is not a uniform improvement but a shift toward predicting hate: the share of items labelled as hate rose for every model. For Qwen-2.5-72B and Gemma-2-27B this lowers the Macro-F1 (79.88% to 78.25% and 76.11% to 73.39%), because most of the extra hate predictions are false positives on this balanced test set. The demonstrations therefore add little to what these two models already do in the zero-shot setting.

#### LLMs performance across fake and real news.

Fig 13 presents the class-wise F1-scores for the Fake News Detection task under both zero-shot and few-shot prompting conditions. Under zero-shot setting, most models obtain the F1-score over 70% in both real and fake classes, which demonstrates they can generalise well without being access to any example with labels. Nonetheless, Mistral-V3-7B is particularly weak at detecting fake news, with an F1-score of only 38.9% for the fake class. The two largest models, Gemma-2-27B and Qwen-2.5-72B, achieve the best performance in both classes, with more than 80% for each. This result demonstrates their superior performance in discriminating between real and fake content using the Bengali language.

Interestingly, the few-shot setting doesn’t give an obvious improvement for this task. Among the smaller models, only Mistral-V3-7B gains a little (fake-class F1 from 38.9% to 45.1%). LLaMA-3.2-3B and DeepSeek-R1-8B become clearly worse, with fake-class F1 falling from 76.8% to 56.2% and from 71.2% to 57.9%. Phi-4-14B shows the largest gain (Macro-F1 from 76.91% to 81.48%), while the two largest models barely move. For instance, Gemma-2-27B exhibits F1-scores of 82.1% and 86.3%, in the case of the fake and real classes consecutively, versus 82.9% and 85.7%, obtained under the zero-shot setup. Similarly, Qwen-2.5-72B shows a marginal increase from 80.5% to 81.2% for the fake class and from 85.4% to 86.2% for the real class. This trend hints that, differently from other tasks, presenting few-shot instances does not largely contribute to the improvement of fake news detection. One possible explanation is that the demonstrations (two fake, three real) mainly shift the models’ prior toward the majority label rather than changing what evidence they use. Across the six models, 91.8% of all few-shot errors are fabricated articles accepted as real, and for LLaMA-3.2-3B the share of fabricated articles that were missed rose from 26.4% to 60.6%.

### Error analysis

This section analyzes the prediction behavior of the evaluated models using confusion matrices. The objective is to understand the common misclassification patterns and assess how model architecture and prompting strategies affect performance across the four Bengali text classification tasks.

#### Best performing models.

The confusion matrices in Fig 14 show the performance of the best-performing models. In the sentiment analysis task, Gemma-2-27B is competitive in both few-shot and zero-shot settings. As shown in Fig 14(a) and 14(b), most samples align along the diagonal, indicating accurate predictions for positive and negative sentiments. Neutral samples labelled as negative make up 23.6% of Gemma-2–27B’s few-shot errors (140 of 594). The most common error, however, runs the other way: positive samples predicted as neutral account for 34.8% of the errors (207 items), followed by positive samples predicted as negative (17.8%).

**Fig 14:**
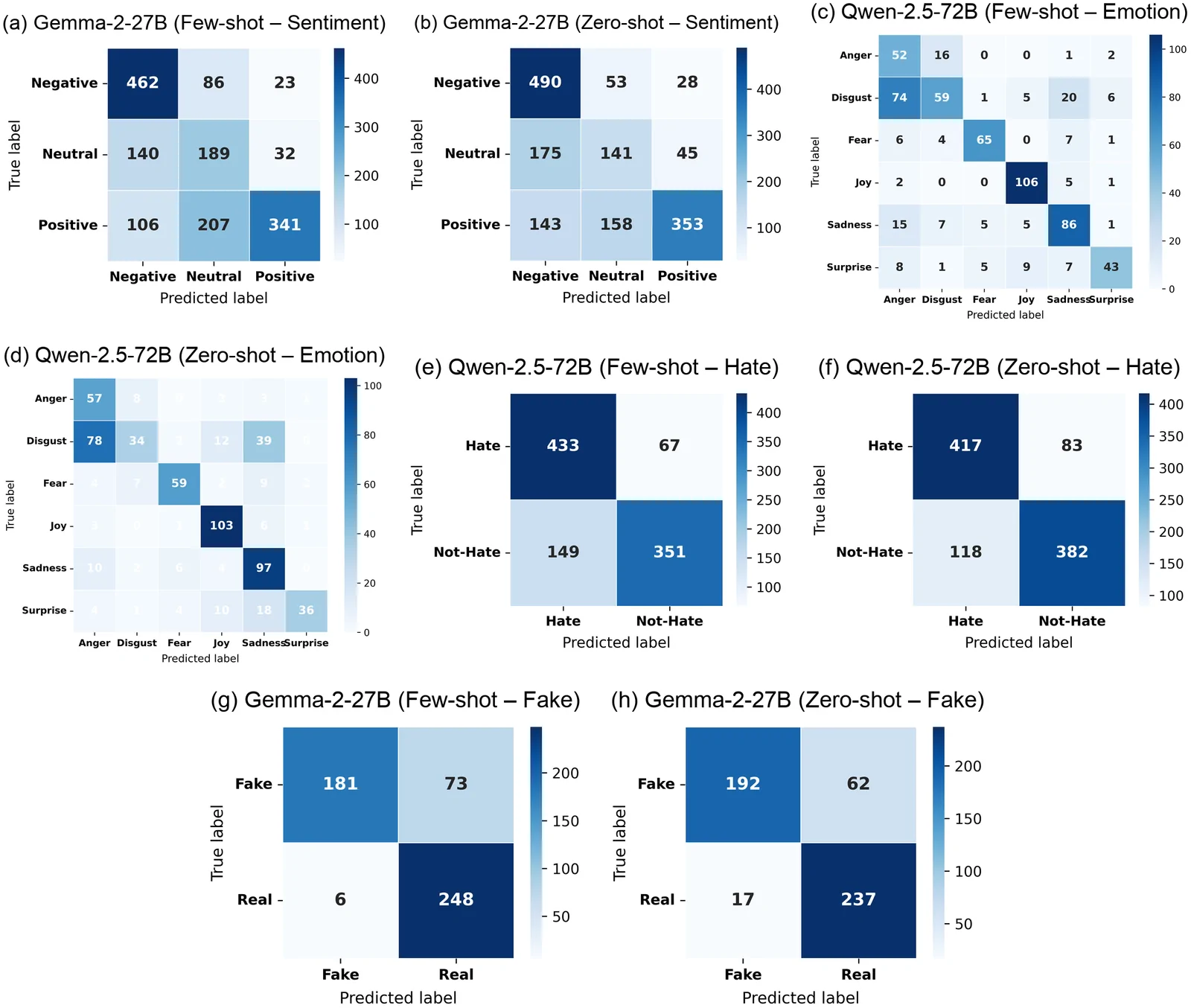
Confusion matrices of top LLMs on Bengali text classification tasks under zero- and few-shot settings. (a) Gemma-2-27B (Few-shot – Sentiment); (b) Gemma-2-27B (Zero-shot – Sentiment); (c) Qwen-2.5-72B (Few-shot – Emotion); (d) Qwen-2.5-72B (Zero-shot – Emotion); (e) Qwen-2.5-72B (Few-shot – Hate); (f) Qwen-2.5-72B (Zero-shot – Hate); (g) Gemma-2-27B (Few-shot – Fake); (h) Gemma-2-27B (Zero-shot – Fake).

For the emotion recognition task, Qwen-2.5-72B demonstrates consistent reliability across major emotion categories, as seen in Fig 14(c) and 14(d). The model is successful in recognising joy and fear, but the identification of anger and disgust appears not to be as straightforward, possibly due to the similar linguistic cues they typically share: disgust items labelled as anger alone make up 34.6% of Qwen-2.5–72B’s few-shot errors (74 of 214), and disgust labelled as sadness another 9.3%. Few-shot prompting does give a small boost in performance for rare emotions such as surprise, which suggests that there is some benefit to seeing a small number of examples from the context.

In the hate speech detection task, Fig 14(e) and 14(f) show that Qwen-2.5-72B achieves balanced classification between hate and not-hate categories. The few-shot setting lowers Qwen-2.5–72B’s false negatives from 83 to 67 but raises its false positives from 118 to 149, so recall for the hate class improves while the Macro-F1 falls slightly. In the fake news detection task, shown in Fig 14(g) and 14(h), Gemma-2-27B achieves high precision for both fake and real content with minimal off-diagonal errors. This consistency demonstrates that the model effectively uses linguistic and contextual cues to separate factual information from misinformation.

Overall, the best-performing models display clear diagonal concentration with comparatively few misclassifications, in line with their higher scores on these benchmarks. Their advantage goes together with larger parameter counts and more multilingual pre-training, but our design cannot separate the two.

#### Worst performing models.

Fig 15 presents the confusion matrices for the least effective models. In sentiment analysis, shown in Fig 15(a) and 15(b), LLaMA-3.2-3B and Mistral-V3-7B display significant confusion between neutral and negative samples, with weak diagonal concentration. This suggests that smaller models lack sufficient semantic depth to capture subtle tonal shifts in Bengali sentiment expressions.

**Fig 15:**
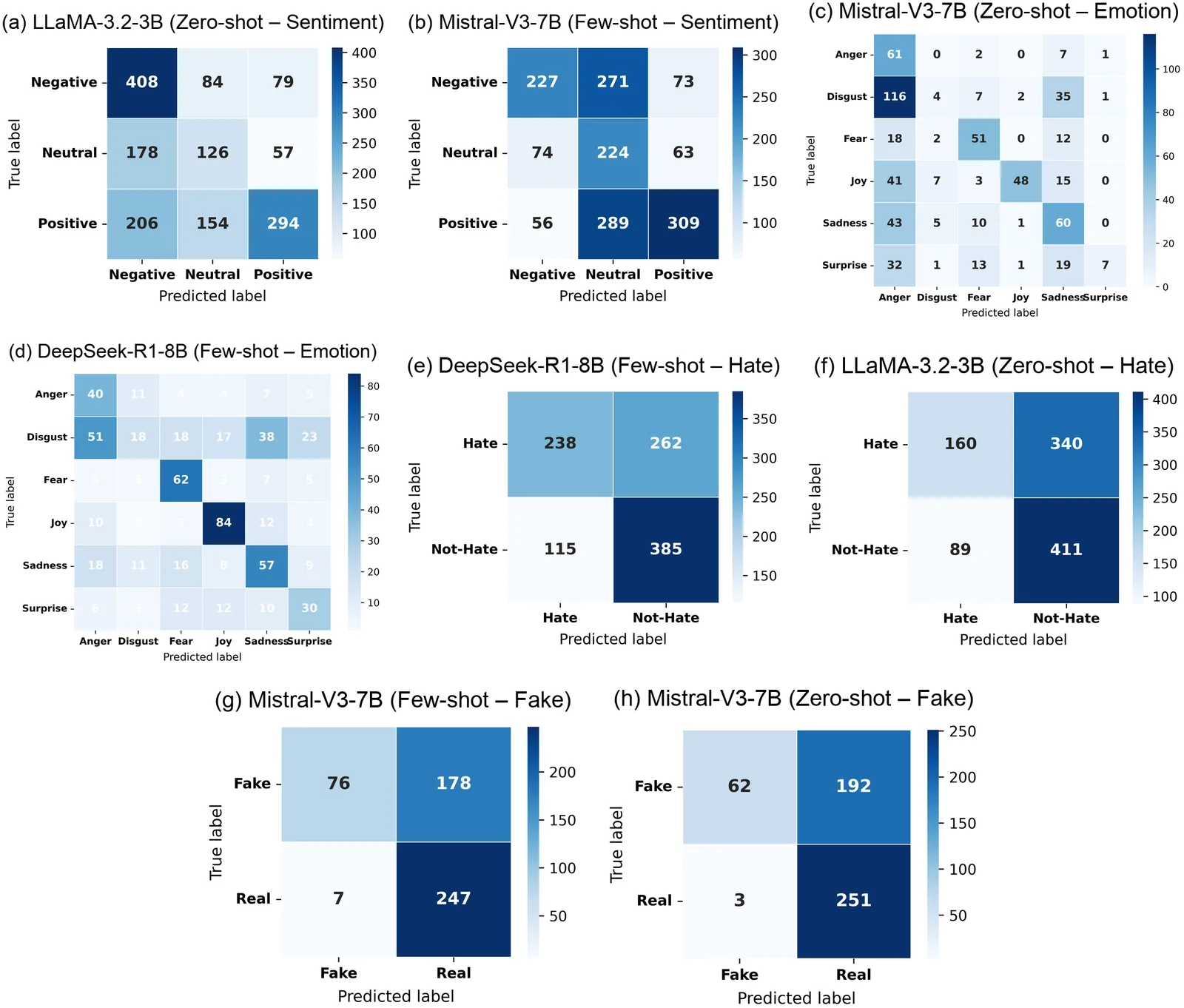
Confusion matrices of low-performing LLMs showing misclassification patterns in Bengali tasks. (a) LLaMA-3.2-3B (Zero-shot – Sentiment); (b) Mistral-V3-7B (Few-shot – Sentiment); (c) Mistral-V3-7B (Zero-shot – Emotion); (d) DeepSeek-R1-8B (Few-shot – Emotion); (e) DeepSeek-R1-8B (Few-shot – Hate); (f) LLaMA-3.2-3B (Zero-shot – Hate); (g) Mistral-V3-7B (Few-shot – Fake); (h) Mistral-V3-7B (Zero-shot – Fake).

For emotion recognition, Fig 15(c) and 15(d) show that Mistral-V3-7B and DeepSeek-R1-8B produce scattered distributions with frequent overlaps between sadness, disgust, and anger. The few-shot configuration helps Mistral-V3-7B significantly (Macro-F1 from 36.08% to 45.18%) but leaves DeepSeek-R1-8B essentially where it was (44.08% to 45.85%, Holm-adjusted *p* = 1.0). Demonstrations thus help some of the small models, but they do not close the gap to the larger ones.

In the hate speech detection task (Fig 15(e) and 15(f)), both DeepSeek-R1-8B and LLaMA-3.2-3B misclassify many hateful statements as not-hate. This pattern suggests that the models may be relatively conservative when identifying hate speech, leading to a higher number of false negatives. In numbers, false negatives make up 79.3% of LLaMA-3.2–3B’s zero-shot errors (340 of 429) and 84.1% of DeepSeek-R1-8B’s (339 of 403). For the large models the picture is reversed: false negatives are only 23.0% of Gemma-2–27B’s zero-shot errors and 41.3% of Qwen-2.5–72B’s. The confusion matrices alone cannot tell us why; the next subsection looks at several possible factors. Finally, for fake news detection, shown in Fig 15(g) and 15(h), Mistral-V3-7B struggles to detect fabricated content, frequently identifying fake instances as real. These scattered prediction patterns demonstrate the limited generalization capability of smaller or less optimised models.

### Qualitative and quantitative error-category analysis

While confusion matrices provide a quantitative overview of model performance, a manual examination of misclassified instances revealed several recurring linguistic challenges in Bengali text classification. The qualitative observations below come from a manual reading of 15 randomly chosen misclassified items per task, taken from the few-shot predictions of Qwen-2.5-72B (60 items in all). Because such a sample is small, each observation is followed by a quantitative check computed over all predictions of all six models.

First, the models frequently struggled with sarcasm and implicit sentiment. For example, expressions containing seemingly positive lexical items were often used sarcastically, causing the models to rely on surface-level word associations rather than the intended pragmatic meaning. This phenomenon was particularly evident in the Emotion Recognition and Sentiment Analysis tasks, where sarcastic or ironic statements were commonly confused with positive emotions. Sarcasm cannot be detected automatically without dedicated annotation, so this category remains qualitative and we did not measure how often it occurs among the errors.

Second, errors were observed in sentences containing negation within otherwise positive contexts. Bengali speakers often use constructions such as “ তার মতো মানুষ হয় না” to express strong admiration, despite the presence of a negative marker. Several models incorrectly interpreted such statements as negative sentiment, suggesting difficulties in capturing the interaction between negation and contextual meaning. To measure the role of negation, we flagged every test item that contains an explicit negation marker (a standalone না, নেই, নয়, নাই or নহে, or a verb ending in - নি) and compared error rates on items with and without such a marker. In emotion recognition, where 262 of the 625 items carry a marker, every model makes more errors on the negated items, by 6–13 percentage points; under few-shot prompting, for example, Qwen-2.5-72B errs on 40.1% of the negated items against 30.0% of the others, and Gemma-2-27B on 43.9% against 35.3%. In sentiment analysis (606 of 1,586 items with a marker), the two error rates differ by at most 4.1 points for any model (37.6% versus 37.3% for Gemma-2-27B). The negation effect seen in the manual inspection is therefore supported for emotion recognition but not for sentiment analysis.

Third, the Hate Speech Detection task revealed challenges related to informal slang and offensive language. Certain colloquial expressions and mild insults were frequently classified as hate speech despite being used in non-hateful conversational contexts. Conversely, some implicitly abusive or derogatory statements were misclassified as non-hateful because they lacked explicit offensive keywords. These observations suggest that the models often relied on lexical cues rather than broader contextual interpretation. In numbers, the direction of the hate-speech errors depends on the model and on the prompting. Under zero-shot prompting the four smaller models mostly miss hateful items: between 66.9% and 84.1% of their errors are hateful items labelled not-hate. Gemma-2-27B does the opposite, with 77.0% of its errors being false positives, and Qwen-2.5-72B sits in between (41.3% false negatives). Under few-shot prompting, where the demonstrations contain three hate and two not-hate examples, every model labels more items as hate, and false positives make up 61.2% of all errors pooled over the six models.

Fourth, in the Fake News Detection task, many fabricated articles were written using realistic journalistic structures, including named entities, numerical details, quotations, and formal news-reporting language. As a result, several fake news instances were incorrectly classified as real news because the models appeared to rely on surface-level credibility cues rather than verifying factual plausibility. This finding suggests that stylistically convincing misinformation remains challenging even for large-scale LLMs. In numbers, 91.8% of all fake-news errors pooled over the six models under few-shot prompting are fabricated articles accepted as real (92.4% for Gemma-2-27B and 97.5% for Qwen-2.5-72B). The fabricated articles that were missed contain numerals more often than those that were caught: 76.7% versus 63.0% for Gemma-2-27B and 78.5% versus 61.7% for Qwen-2.5-72B under few-shot prompting, and 79.0% versus 63.0% and 82.3% versus 60.0% under zero-shot prompting. This supports the idea that numerical detail works as a credibility cue. Article length and quotation marks, on the other hand, do not: the missed fabrications were not longer than the detected ones (266 versus 297 words on average for Gemma-2-27B) and did not contain quotation marks more often (58.9% versus 65.2%).

Overall, the qualitative analysis indicates that pragmatic language phenomena, including sarcasm, contextual negation, culturally specific slang usage, and the realistic journalistic style of fabricated news articles, constitute important sources of error for current open-weight LLMs in Bengali text classification. The simple counts above (confusion pairs, negation markers, error direction and numeral frequency) support the negation, label-prior and numerical-cue explanations but not the length and quotation explanations. Sarcasm and other pragmatic phenomena could only be judged by hand and would need proper annotation in future work.

### Receiver operating characteristic (ROC-AUC) analysis

In this section, we evaluate the discriminative performance of the models using confusion matrix analysis, ROC curves, and AUC scores. The ROC–AUC score attempts to present a fair judgment to compare models’ ability to distinguish between classes across different prompting strategies. For multiclass classification tasks (e.g., Sentiment Analysis, Emotion Recognition), the One-vs-Rest (OvR) strategy is employed to calculate the area under the ROC curve (AUC), where each class is iteratively treated as the positive class against all others. Fig 16 illustrates the ROC–AUC results for all four Bengali text classification tasks under both zero-shot and few-shot conditions.

**Fig 16:**
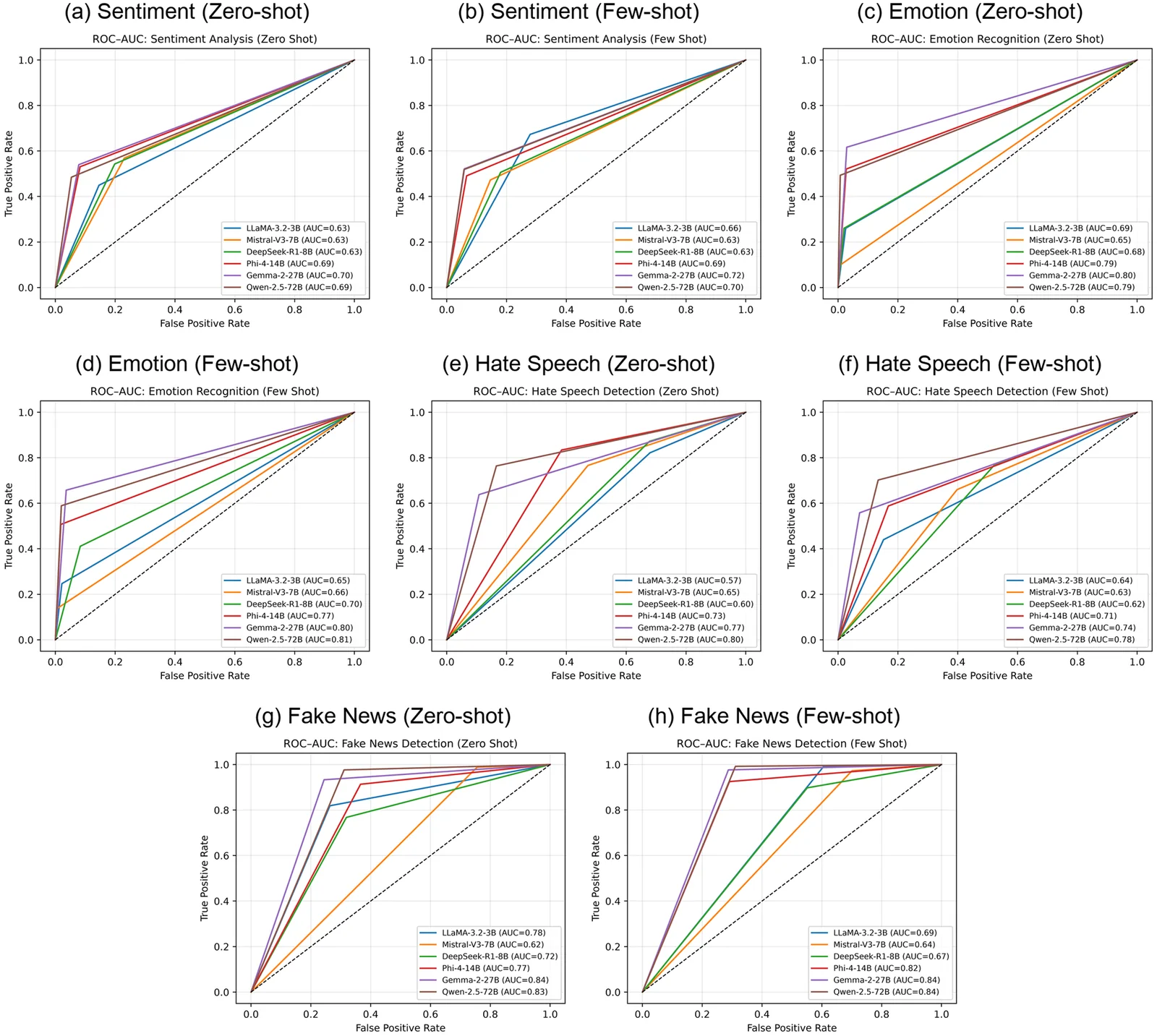
ROC–AUC curves of six LLMs on Bengali text classification tasks under zero- and few-shot settings. (a) Sentiment (Zero-shot); (b) Sentiment (Few-shot); (c) Emotion (Zero-shot); (d) Emotion (Few-shot); (e) Hate Speech (Zero-shot); (f) Hate Speech (Few-shot); (g) Fake News (Zero-shot); (h) Fake News (Few-shot).

As shown in Fig 16(a) and 16(b), the sentiment analysis task reveals poor to acceptable discrimination for the evaluated models, with AUC values ranging from 0.63 to 0.72. Gemma-2-27B achieves the highest AUC of 0.72 in the few-shot setting, followed closely by Qwen-2.5-72B (0.70), demonstrating more confident and stable classification boundaries. Smaller models such as LLaMA-3.2-3B and Mistral-V3-7B show weaker discrimination, indicating difficulties in capturing subtle sentiment cues in Bengali.

For the emotion recognition task (Fig 16(c) and 16(d)), all models get a higher AUC value than that of the sentiment task. As shown in the few-shot setting, Qwen-2.5-72B and Gemma-2-27B achieve higher 0.81 and 0.80 than others, revealing their healthy discriminative power on emotional tone. While Phi-4-14B also maintains a competitive AUC at 0.77, smaller models fail to consistently capture sensitivity between groups of emotion classes.

In hate speech detection (Fig 16(e) and 16(f)), Gemma-2-27B and Qwen-2.5-72B outperform the other ones with few-shot AUCs higher than 0.74 and 0.78, respectively. This improvement highlights the benefit of in-context learning for sensitive social classification tasks where context and tone are important factors. By contrast, LLaMA-3.2-3B gets a low AUC of only 0.57 in the zero-shot setting, indicating that LLaMA-3.2-3B is less strong in detecting offensive or hateful content.

The fake news detection task (Fig 16(g) and 16(h)) obtains the highest AUC values in all tasks. Gemma-2-27B reaches 0.84 in both settings, and Qwen-2.5-72B 0.83 under zero-shot and 0.84 under few-shot prompting, a good level of class-balanced discrimination between real and fake text. This agrees with the higher fake news scores of the two largest models, although these data cannot tell whether the advantage comes from factual knowledge or from stylistic cues.

## Discussion

This section looks at the results in the light of the statistical analyses; Fig 17 gives the overall picture. Three findings hold up after correction for multiple comparisons. First, the six models differ significantly on every task and in every setting (Cochran’s Q, all *p* < 10^–25^), and the two largest models, Qwen-2.5-72B and Gemma-2-27B, have the highest Macro-F1 on every task. Second, the ranking does not follow model size. Gemma-2-27B is ahead of Qwen-2.5-72B on sentiment analysis (significantly so under zero-shot prompting) and on fake news detection; LLaMA-3.2-3B cannot be separated from Phi-4-14B on zero-shot fake news detection; and the 7B Mistral and 8B DeepSeek models swap places depending on the task, Mistral-V3-7B being significantly better on zero-shot hate speech detection and DeepSeek-R1-8B on zero-shot emotion recognition and fake news detection. Third, the effect of our single five-shot demonstration set is small and uneven. After Holm correction it is significant for only four of the 24 model–task combinations: three improvements (LLaMA-3.2-3B on sentiment analysis and hate speech detection, Mistral-V3-7B on emotion recognition) and one loss (LLaMA-3.2-3B on fake news detection). It is never significant for Phi-4-14B, Gemma-2-27B or Qwen-2.5-72B. The difficulty of the tasks is the same for all models and settings: fake news detection gives the two largest models their highest Macro-F1 (82.91–84.33%), while emotion recognition and sentiment analysis are the hardest tasks (best Macro-F1 66.80% and 60.68%).

**Fig 17:**
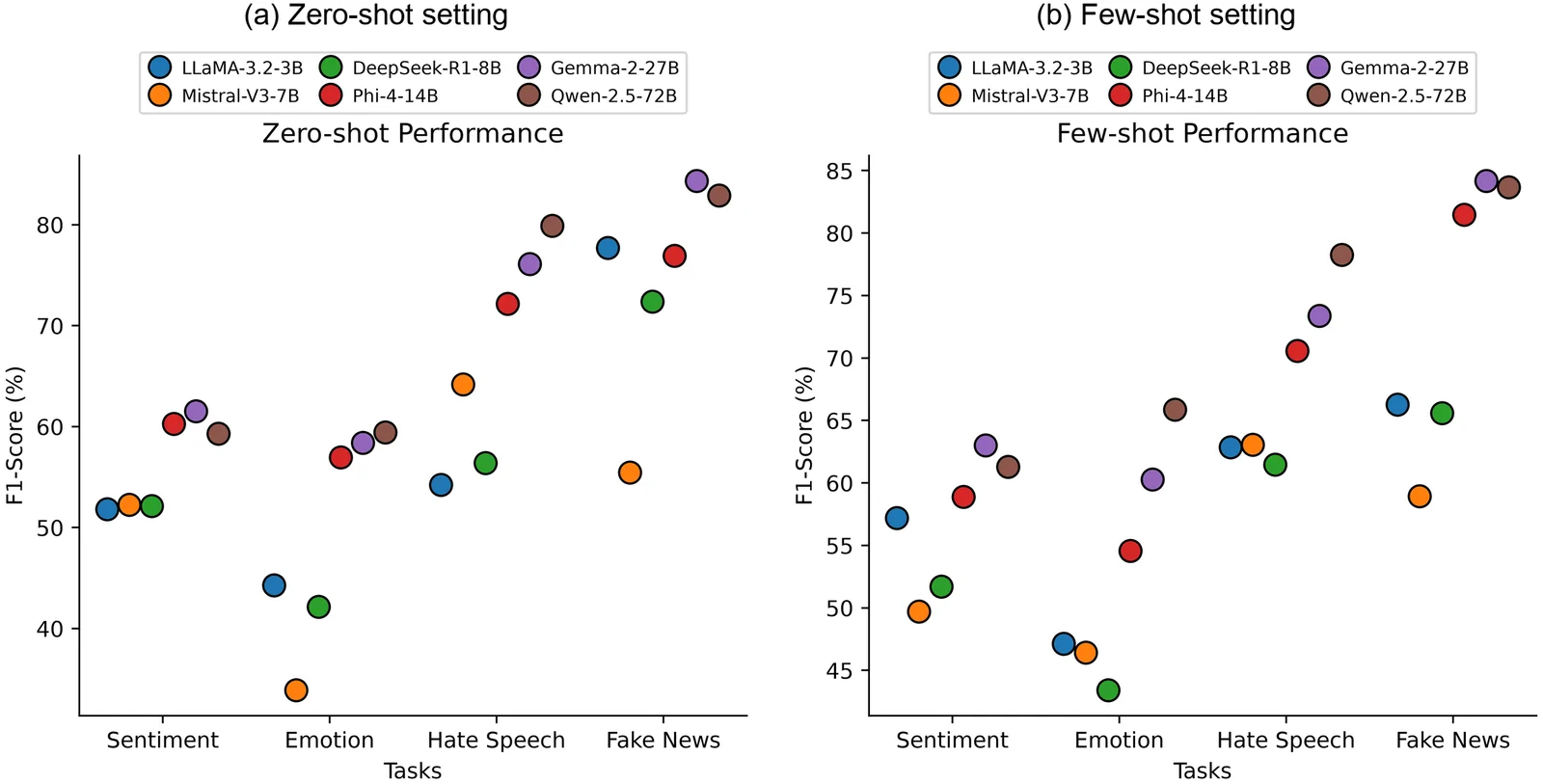
LLMs’ performance with two prompt settings across various classification tasks. (a) Zero-shot setting. (b) Few-shot setting.

Why the largest models do best cannot be settled with this design, because model size and model family go together. Each size is represented by exactly one family, and the families differ in many things at once: pre-training corpus and language coverage, tokenizer vocabulary (32,768 tokens for Mistral-V3-7B, 100,352 for Phi-4-14B, 128,256 for LLaMA-3.2-3B and DeepSeek-R1-8B, and roughly 152K and 256K for Qwen-2.5-72B and Gemma-2-27B), instruction tuning and alignment, and, for Qwen-2.5-72B, 4-bit AWQ quantization. The within-family comparison that would isolate size (for example Qwen-2.5 at 7B, 14B, 32B and 72B) was not run. Two observations nevertheless speak against parameter count as the only explanation. Gemma-2-27B, trained mostly on English data, matches or beats the 72B Qwen model on two of the four tasks. And DeepSeek-R1-8B, which shares the Llama-3 tokenizer and architecture with LLaMA-3.2-3B but was post-trained for mathematical and code reasoning, is never significantly more accurate than the 3B model, and is significantly less accurate on few-shot sentiment analysis. Post-training objectives and language coverage therefore seem to matter at least as much as size. For this reason we describe the advantage of the largest models as an association with scale and multilingual pre-training, not as an effect of size (RQ2).

Differences in model behaviour are also evident at the task level. Sentiment analysis is relatively simple yet only achieves moderate results: the best Macro-F1 is 60.68% (Gemma-2-27B, few-shot), even though accuracy reaches 62–63%. The confusion pairs show that the main problem is not confusion between neutral and negative but a drift toward the neutral label. Pooled over the six models under few-shot prompting, positive items predicted as neutral make up 29.6% of all sentiment errors and negative items predicted as neutral another 20.7%, while neutral items predicted as negative make up 17.5%. It should also be noted that the SentiNoB dataset exhibits class imbalance, containing 654 positive samples, 571 negative samples, and only 361 neutral samples. Therefore, the relatively lower F1-scores observed for the neutral class may be influenced not only by model limitations but also by the unequal class distribution. The reduced representation of neutral samples may make it more difficult for LLMs to accurately identify the characteristics of this category. Consequently, class imbalance should be considered a potential contributing factor when interpreting neutral-class performance. The emotion recognition still remains the most challenging task, while the state-of-the-art method yields 66.80% Macro-F1 in the few-shot scenario. This limitation might be due to the fine-grained nature of our task where we have to discriminate between six very similar feelings that often share overlapping characteristics in Bengali. A single confusion pair, disgust predicted as anger, accounts for 26.2% of all emotion errors pooled over the six models (49.0% of Gemma-2–27B’s and 34.6% of Qwen-2.5–72B’s few-shot errors). Disgust is also the largest class in the test set (165 of 625 items) and anger the smallest (71), and the few-shot prompt contains no anger example; in line with this, the anger-class F1 fell under few-shot prompting for four of the six models, including the three strongest. Negation is a second measurable factor: items with an explicit negation marker are misclassified 6–13 percentage points more often than other items by every model.

Results of hate speech detection are more promising, with state-of-the-art approaches approaching 80% F1-score. The binary nature of the task, and better linguistic cues for hate speech make it relatively easier. The direction of the errors depends on the model and the setting, so the models are not uniformly conservative. Under zero-shot prompting the four smaller models mostly miss hateful items (66.9–84.1% of their errors are hateful items labelled not-hate), whereas Gemma-2-27B mostly over-flags (77.0% of its errors are false positives). Under few-shot prompting, the demonstration set with three hate and two not-hate examples pushed every model toward predicting hate, and false positives make up 61.2% of all pooled errors. For content moderation this means that the same model can be too lenient or too strict depending on the prompt, which argues for calibrating on deployment data rather than relying on benchmark scores. Fake News detection is the most tractable task, with the best-performing models achieving F1-scores over 84%. This high accuracy is most likely due to the clear stylistic and factual differences between real and fake articles. Still, 91.8% of the pooled fake-news errors are fabricated articles accepted as real, and the missed fabrications contain numerals more often than the detected ones (76.7% versus 63.0% for Gemma-2-27B), which points to reliance on surface credibility cues. In addition, the evaluation set is balanced while fabricated articles make up only about 1.3% of the source corpus, so the fake-class precision reported here would not carry over to the natural class distribution.

The few-shot effect depends on the task and the model, and for the strongest models it cannot be told apart from zero (Table 8). Where the demonstrations help, they help the weakest model on the task (LLaMA-3.2-3B on sentiment analysis and hate speech detection, Mistral-V3-7B on emotion recognition), which suggests that they mainly fix problems with the task format or the label prior rather than adding knowledge of Bengali. Where they hurt (LLaMA-3.2-3B on fake news detection, whose miss rate on fabricated articles rose from 26.4% to 60.6%), the demonstration set seems to have pulled the model toward the majority label of the demonstrations (three real, two fake). Our answer to RQ3 is therefore a cautious one: with one fixed demonstration set, five-shot prompting improves classification performance under the evaluated Bengali task settings for some of the smaller models and does not measurably change it for the larger ones. Since the choice, order and label balance of demonstrations are known to change few-shot results considerably, and we tested only one configuration, neither the size nor the direction of these effects should be generalised to five-shot prompting as such.

A further consideration concerns the absence of comparisons with Bengali-specific pre-trained language models, such as BanglaBERT, and proprietary frontier LLMs, such as GPT-4. Recent studies have emphasized the importance of incorporating strong baseline models to provide a more comprehensive and rigorous evaluation of proposed approaches [56–60]. In the present study, our primary objective was to conduct a systematic comparison of contemporary open-weight instruction-tuned LLMs under identical zero-shot and few-shot prompting settings. Consequently, the experimental design focused exclusively on publicly available open-weight models. Furthermore, BanglaBERT is typically evaluated under supervised fine-tuning settings, whereas our work investigates the in-context learning capabilities of generative LLMs without task-specific parameter updates. Therefore, a direct comparison would not constitute a fully equivalent evaluation setting. Nevertheless, we acknowledge that including Bengali-specific encoder models and proprietary frontier LLMs would provide additional insights into the relative strengths and limitations of open-weight LLMs. Future work should incorporate such baselines to further strengthen benchmarking efforts for Bengali NLP tasks.

Finally, benchmark performance should not be confused with real-world effectiveness. All results come from four test sets that are small (508–1,586 items), largely drawn from social media and, in two cases, artificially balanced. The confidence intervals in Table 9 are 4.7–9.0 Macro-F1 points wide, and several neighbouring models cannot be told apart statistically (for example Phi-4-14B and Gemma-2-27B on zero-shot emotion recognition and on few-shot fake news detection). The scores therefore rank the models under the prompting conditions we evaluated, but they do not show that any model is ready for deployment, where class priors, text genres, dialects and adversarial behaviour all differ from the benchmarks. What the data do support are a few practical regularities: among the models tested, the largest open-weight models are the most reliable choice for Bengali classification; demonstrations are no substitute for calibration on the target label distribution; and the remaining errors concentrate on a few identifiable phenomena (neutral drift in sentiment analysis, the disgust–anger boundary and negation in emotion recognition, the prompt-induced label prior in hate speech detection, and numeral-rich fabrications in fake news detection) that could be targeted with calibration, parameter-efficient fine-tuning, or retrieval of factual evidence.

### Implications for low-resource language NLP

Findings from this research study carry several crucial results for furthering NLP in languages with low-resources like Bengali. First, the wide gap in performance between large-scale models (Qwen-2.5-72B and Gemma-2-27B) and small ones (LLaMA-3.2-3B and Mistral-V3-7B) shows that, among the open-weight models available today, the largest ones are the most reliable choice for Bengali text classification. This association cannot be put down to parameter count alone, however, because size and model family go together (see above). But this also results in an accessibility challenge, as it remains a significant obstacle for many researchers and institutions working in resource-constrained environments the computational and hardware requirements of big models.

Second, the uneven and mostly non-significant effect of a single five-shot demonstration set shows that demonstrations alone do not guarantee gains. A few labelled examples help mainly the weaker models, and mainly by fixing the output format and the label prior; larger gains from limited labelled data will probably require calibration or parameter-efficient fine-tuning. This finding is particularly valuable for languages without large annotated datasets, where creating large corpora is difficult or expensive. With the creation of small but high-quality labelled datasets, future research in Bengali and other similar languages should move away from only looking at developing huge corpora. Such small datasets could serve both as pools of demonstrations, whose selection should be checked over several demonstration sets, and as data for calibration or fine-tuning.

Thirdly, differences in task difficulty demonstrate that the choice of method and problem framing significantly affect model performance. Tasks with clear-cut categorical distinctions–-e.g., hate speech and fake news detection reach the highest benchmark scores with existing open-weight LLMs, although the error analysis shows that these scores depend on the label prior that the prompt induces. In contrast, fine-grained tasks like emotion recognition, which require thinking deeply about subtle expressions and environmental cues, might profit from different methods such as fine-tuning, ensemble learning or hybrids of LLMs with task-specific classifiers. The development of adaptive methods that balance generalisation against linguistic sensitivity could make models more robust in complex low-resource language environments.

### Limitations

While this study provides valuable insights into the performance of open-weight LLMs for Bengali text classification, several limitations must be acknowledged. First, our evaluation is restricted exclusively to classification tasks. Although classification is a fundamental component of NLP, future research should expand this analysis to include generative tasks such as text summarization, machine translation, and question answering to draw more comprehensive conclusions about LLM capabilities in Bengali.

Second, to keep the study transparent and reproducible, only open-weight models were evaluated. However, this selection excludes the comparison to closed-source proprietary systems (such as GPT-4 or Claude), which might have a better overall performance. They should make it to future benchmarks — providing a better perspective of how wide the open-closed ecosystem gap is for Bengali language processors.

Third, the prompting method adopted here is exclusive to zero-shot and few-shot (five-shot) settings. More advanced methods of prompting (e.g., chain-of-thought reasoning, self-consistency or retrieval-augmented generation) could lead to more improvements but were not in the scope of this work. Also, parameter-efficient fine-tuning mechanisms such as LoRA or QLoRA, could be pursued and provide practical ways to improve the performance of smaller models without spending much in terms of computing time. Moreover, all instructions were written in English while the inputs were in Bengali. We did not vary this; Bengali or bilingual instructions may interact differently with the instruction-tuning data of each model, and the language of the prompt should be treated as an experimental factor in future work. Furthermore, the few-shot condition used one fixed set of five demonstrations per task, in one order, with one random seed. The choice, order and label balance of demonstrations are known to affect in-context learning, and our own results show that the demonstrations shifted the label prior for hate speech and fake news detection. The few-shot effects reported here are therefore estimates for this particular demonstration set, and they would need to be replicated over several demonstration sets before being generalised.

Fourth, the datasets we used, while standard benchmarks in the Bengali NLP community, suffer from natural limitations in terms of size and coverage, domain diversity, and annotation consistency. For instance, much of the data originates from social media platforms and may not accurately reflect formal or domain-specific Bengali. In addition, some datasets are small (e.g., the emotion recognition corpus: only 625 examples), which may compromise statistical stability and the generalisation value of the results. Furthermore, the SentiNoB dataset exhibits class imbalance, containing 654 positive samples, 571 negative samples, and only 361 neutral samples. This imbalance may have influenced class-wise performance, particularly the lower F1-scores observed for the neutral category in the sentiment analysis task. Conversely, the fake news evaluation set was balanced by downsampling real news to a 1:1 ratio, whereas fabricated articles make up only about 1.3% of the source corpus. The scores for this task therefore describe performance under an artificial class prior and should not be read as real-world detection effectiveness.

Fifth, model size and model family go together in our design. Each size corresponds to a single family with its own pre-training corpus, tokenizer, instruction-tuning recipe and, for Qwen-2.5-72B, quantization scheme, and we did not evaluate several sizes of the same family. Statements about the role of model size are therefore about association only; isolating size would require several sizes of one family evaluated under identical conditions.

Sixth, the statistical analysis has limits of its own. Each configuration was run once with deterministic settings, so run-to-run variance is not captured. The pairwise permutation tests involve only Qwen-2.5-72B and Gemma-2-27B, and the multi-model analysis works on per-item correctness (accuracy) rather than on Macro-F1. The quantitative error analysis relies on simple, automatically computable indicators (negation markers, numerals, error direction), whereas sarcasm and other pragmatic phenomena were judged only on a small, manually inspected sample.

Finally, although we focus on quantitative performance measures (i.e., accuracy or F1-score) in this work, we do not delve into more holistic notions of fairness, bias, and cultural relevance of model predictions. Because of the sensitive nature of tasks like hate speech detection and fake news classification, future work must also include bias assessment frameworks to analyse fine-grained model behaviour in different demographic groups, social context, and dialectic variation within the Bengali-speaking community.

## Conclusion

A comprehensive evaluation of six open-weight Large Language Models (LLMs)–-LLaMA-3.2-3B, Mistral-V3-7B, DeepSeek-R1-8B, Phi-4-14B, Gemma-2-27B, and Qwen-2.5-72B is presented with this research, where four Bengali text classification tasks are evaluated, such as sentiment analysis, emotion recognition, hate-speech detection, and fake-news detection. For ensuring equal and consistent comparisons, both zero-shot and few-shot prompting strategies were applied using both the vLLM inference engine and Hugging Face Transformers pipelines. The two largest models, Qwen-2.5-72B and Gemma-2-27B, obtained the best scores on all four tasks, and paired multi-model tests with Holm correction confirmed that the six models differ significantly. The ranking did not follow parameter count, however, and because model size and model family go together in this study, the results do not establish that size itself causes the difference. With a zero-shot setting, the best-performing model on hate-speech detection is Qwen-2.5-72B with 79.90% accuracy and a Macro-F1 score of 79.88%, whereas for fake-news classification, 84.45% accuracy is achieved with Gemma-2-27B as well as an 84.33% Macro-F1 score. After a few-shot prompt was applied, Gemma-2-27B maintained strong performance on fake-news detection with a Macro-F1 score of 84.17%. Qwen-2.5-72B achieved the best emotion-recognition result, reaching a Macro-F1 score of 66.80%. Five-shot prompting with a single fixed demonstration set changed the results significantly for only four of the 24 model–task combinations, and never for the three largest models. Overall, the results describe benchmark classification performance under the prompting conditions we evaluated rather than real-world effectiveness. They indicate that model scale and multilingual pre-training go together with stronger Bengali text classification, while in-context demonstrations mainly help the weaker models and shift the label prior.

### Future work

Though the findings are pretty favourable, several issues still need careful attention. A difficulty is seen among the models that are working to classify the neutral as well as the negative statement, and they also frequently lump together emotions with similar meanings, such as anger and disgust, as well as misleading statements, which are also labelled as real. These mechanisms can be strengthened further in the future. For improving the ability of the model, approaches that are improved should be applied, such as step-by-step reasoning or the use of external information sources. With an intention to improve the performance, along with the requirement of not using expensive hardware, Lightweight fine-tuning approaches such as LoRA or QLoRA can be applied. In order to acquire an improved awareness of their overall capabilities and limits, a broader set of NLP tasks, for instance, text generation, summarisation, and question answering, should be tested as well. Additionally, retaining the NLP system, which is a blessing for Bangali users, should pay great attention to its fairness across different social and demographic groups. With a view to improving further, larger and more diverse datasets should be introduced, which could be a great help to Bangali NLP tool users.
